# Spherically Restricted Random Hyperbolic Diffusion

**DOI:** 10.3390/e22020217

**Published:** 2020-02-14

**Authors:** Philip Broadbridge, Alexander D. Kolesnik, Nikolai Leonenko, Andriy Olenko, Dareen Omari

**Affiliations:** 1 Department of Mathematics and Statistics, La Trobe University, Melbourne VIC 3086, Australia; P.Broadbridge@latrobe.edu.au (P.B.); omari.d@students.latrobe.edu.au (D.O.); 2 Institute of Mathematics and Computer Science, Academy Street 5, 2028 Kishinev, Moldova; kolesnik@math.md; 3 School of Mathematics, Cardiff University, Senghennydd Road, Cardiff CF24 4AG, UK; leonenkon@cardiff.ac.uk

**Keywords:** stochastic partial differential equations, hyperbolic diffusion equation, spherical random field, Hölder continuity, long-range dependence, approximation errors, cosmic microwave background

## Abstract

This paper investigates solutions of hyperbolic diffusion equations in $R^{3}$ with random initial conditions. The solutions are given as spatial-temporal random fields. Their restrictions to the unit sphere $S^{2}$ are studied. All assumptions are formulated in terms of the angular power spectrum or the spectral measure of the random initial conditions. Approximations to the exact solutions are given. Upper bounds for the mean-square convergence rates of the approximation fields are obtained. The smoothness properties of the exact solution and its approximation are also investigated. It is demonstrated that the Hölder-type continuity of the solution depends on the decay of the angular power spectrum. Conditions on the spectral measure of initial conditions that guarantee short- or long-range dependence of the solutions are given. Numerical studies are presented to verify the theoretical findings.

## 1. Introduction

Numerous environmental, biological and astrophysical applications require the modelling of changes in data on the unit sphere $S^{2}$ or in the 3D space $R^{3}$ [1,2,3,4,5,6]. One of the conventional tools for such modelling is stochastic partial differential equations (SPDEs)—see, for example [1,5,7,8], and the references therein. Random fields that are solutions of such SPDEs often exhibit dynamics dependent on initial conditions. Investigating the properties of these random fields is important for theoretical insight and practical applications.

SPDEs on surfaces and Riemannian manifolds have found numerous applications to problems in cosmology, physics, biology, fluid dynamics and pattern formation on surfaces, just to mention a few. See [7,9,10,11] and the references therein.

Random fields on a sphere, one of the simplest two-dimensional manifolds, have been used as a standard model in the astrophysical and cosmological literature in the last several decades [3,4,8,12]. NASA and ESA space missions [4] obtained very detailed measurements of Cosmic Microwave Background radiation (CMB), which are interpreted as a realisation of a spherical random field superimposed on an underlying signal of large-scale acoustic waves in plasma near the time of recombination. The theory of the standard inflation scenario uses a Gaussian model for the density fluctuation of this field [3,4,6]. Several new cosmological models were proposed using non-Gaussian assumptions and employed sophisticated statistical tests to justify possible departures from Gaussianity. The understanding of changes in CMB temperature fluctuations is important to predict future cosmological evolution and accurately reconstruct past states of the Universe. It also can help in the estimation and statistical inference of physical parameters obtained from the CMB data. SPDEs on the sphere can be used to model changes in CMB temperature fluctuations; see [7,8].

The pronounced spectral peaks at very large wavelengths in CMB temperature data are evidence of acoustic waves that were seeded by earlier superluminal inflation, with remnant coherent waves remaining until last scattering of photons and recombination of atoms around 340,000 years after the big bang [6,13]. In the plasma Universe there was chaotic mixing, but it is problematic to represent the underlying particle kinematics as standard Brownian motion, which leads to the standard diffusion equation for particle densities. Under standard diffusion, density disturbances have unbounded propagation speeds, which is unacceptable in relativistic cosmological contexts wherein remnant structures that are coherent over space-like domains have not been smeared away by diffusion. Reimberg [14] has directly modelled a sequence of photon–electron collisions backwards in time from the last scattering, with random changes of direction, and with the same distance travelled over equal time steps—unlike in usual random flight theory. Giona has developed a Feynman–Kac stochastic dynamics by which a particle undergoes a succession of collisions with speed-limited jumps. Consequently, the diffusion coefficients decrease as a power of the Lorentz–Fitzgerald contraction factor of an inertial observer. This has the interesting consequence that different observers may disagree on whether a process is deterministic or stochastic. Non-trivial continuous relativistic Markov processes on position space are simply not possible [15]. A simpler alternative phenomenological model is effected by replacing the standard diffusion equation by the simplest hyperbolic diffusion equation that has a variable but bounded speed of propagation. Ali and Zhang [16] recast the hyperbolic diffusion equation as a Lorentz-invariant Liouville conservation equation in one time and four space dimensions, before restricting $x^{4}$ to be ict. Ali and Zhang then retain the second law of thermodynamics but only as a reaction-diffusion equation in 1+4 dimensions. Section 8 shows how information entropy may decrease by a small amount during the propagation of a point source by hyperbolic diffusion, whereas the overall increase is much larger.

The so-called Cattaneo hyperbolic diffusion equation [17,18] has been used to explain outcomes of heat conduction experiments in liquid $He^{4}$ in the super-fluid state [19,20], and solid $He^{3}$ and solid $He^{4}$ at very low temperatures. In these materials, and in some nanotubes and other graphite structures [21], heat energy propagates as a “second sound” wave mediated by phonons, at a propagation speed of around one-tenth the normal speed of sound. Since the experiments on graphite have been conducted on nano-scale samples, we expect that waves of second sound could likewise be detected in the spherical surface of a $C^{60}$ or larger fullerene ball, as formulated in our previous paper on hyperbolic diffusion on a spherical surface [8].

SPDEs on $R^{3}$ have been extensively studied. However, SPDEs on manifolds have only recently attracted a great deal of attention [7,8,22,23]. The results in these papers demonstrate that the continuity properties of solutions and the convergence rates of approximations to solutions are determined by decay rates of the angular power spectrum of initial random conditions. This article continues studies of solutions of SPDEs on the sphere. However, in contrast to the above publications that directly model spherical random fields using Laplace or Laplace–Beltrami operators on the sphere, we employ another approach. Namely, we consider the restriction of the stochastic hyperbolic diffusion in $R^{3}$ to the unit sphere. Compared to the available literature this approach is more consistent with real CMB observations that exist in 3D space but are measured only on $S^{2}$. From a mathematical point of view, additional investigations are required to show that solutions of known models on the sphere admit physically meaningful extensions to $R^{3}$ that are consistent with 3D observations. By its construction, our model directly provides this consistency. The proposed model may find new applications for the next generation of CMB experiments, CMB-$S^{4}$, which will be collecting 3D observations. A very detailed discussion of SPDEs on manifolds and their physical and mathematical justification for CMB problems can be found in [8]. The hyperbolic diffusion equation prohibits the superluminal propagation of density disturbances, which is an unwanted feature of pure diffusion models over super-galactic distances. In addition, the linear hyperbolic diffusion equation, expressed in terms of co-moving material space coordinates and conformal time coordinate, is a good approximation to the field equation of a scalar field minimally coupled to an expanding Robertson–Walker space-time. However, speed-limited diffusion raises some interesting questions about the dynamics of Shannon entropy. For physical concentrations governed by linear or nonlinear heat diffusion equations of parabolic type, Shannon entropy is fully analogous to thermodynamic entropy and it increases monotonically [24]. It will be explained that at low wave numbers, the hyperbolic diffusion equation behaves as a dissipative diffusion equation. However, above some cut-off wave number it behaves as a bi-directional wave equation which has increasing entropy when twin pulses separate but has decreasing entropy when pulses approach each other constructively.

This paper is organised as follows. Section 2 presents definitions and results about spatial-temporal random fields in $R^{3}$. It also introduces hyperbolic diffusion equations with random initial conditions and their solutions. Section 3 investigates the spatial-temporal hyperbolic diffusion field on the unit sphere. The Hölder-type continuity of the exact solution of the spatial-temporal hyperbolic diffusion field on the sphere is investigated in Section 4. In Section 5 we study the dependence structures of the spherical hyperbolic diffusion random fields. Section 6 obtains the mean-square convergence rate to the diffusion field in terms of the angular power spectrum. Section 7 provides some numerical results. Finally, Shannon entropy behaviour is discussed in Section 8, followed by some conclusions.

In the following sections we will use the symbol C to denote constants that are not important for our exposition. The same symbol may be used for different constants appearing in the same proof.

## 2. Spatial Random Hyperbolic Diffusion

This section reviews the basic theory of random fields in $R^{3}$ and introduces a hyperbolic diffusion with random initial conditions. Then, the solution of the diffusion equation is derived and analysed.

We consider the hyperbolic diffusion equation

(1) $$\frac{1}{c^{2}}\frac{\partial^{2}q\left(x,t\right)}{\partial t^{2}}+\frac{1}{D}\frac{\partial q(x,t)}{\partial t}=\Delta q\left(x,t\right),$$

$$x=\left(x_{1},x_{2},x_{3}\right)\in R^{3},\;t\geq 0,\;D>0,\;c>0,$$

where q(x,t)=q(x,t,ω),ω∈Ω, is a random field satisfying the random initial conditions:

(2) $${q\left(x,t\right)|}_{t=0}=\eta \left(x\right),\;{\left.\frac{\partial q(x,t)}{\partial t}\right|}_{t=0}=0,$$

where Δ is the Laplacian in $R^{3}$ and the random field $\eta \left(x\right)=\eta \left(x,\omega \right),\;x\in R^{3},\;\omega \in \Omega$, defined on a suitable complete probability space (Ω,F,P), is assumed to be measurable, mean-square continuous, wide-sense homogeneous and isotropic with zero mean and the covariance function B(∥x−y∥)=Cov(η(x),η(y)).

The covariance function has the following representation:

$$B(∥x-y∥)=\int_{R^{3}}\cos\left(\langle \kappa ,x-y\rangle \right)\;F\left(d\kappa \right)=\int_{0}^{\infty }\frac{\sin(\mu ∥x-y∥)}{\mu ∥x-y∥}\;G\left(d\mu \right),$$

for some bounded, non-negative measures F(·) on $\left(R^{3},B\left(R^{3}\right)\right)$ and G(·) on $\left(R_{+}^{1},B\left(R_{+}^{1}\right)\right)$, such that

$$F\left(R^{3}\right)=G\left(\left[0,\infty \right)\right)=B\left(0\right),\;G\left(\mu \right)=\int_{\left\{∥\kappa ∥<\mu \right\}}F\left(d\kappa \right).$$

See [25], pp. 1–5 and [26], pp. 10–15 for more details.

Then, there exists a complex-valued orthogonally scattered random measure Z(·) such that for every $x\in R^{3}$, the field η(x) itself has the spectral representation

(3) $$\eta \left(x\right)=\int_{R^{3}}e^{i\langle \kappa ,x\rangle }\;Z\left(d\kappa \right),\;\;E{\left|Z\left(\Delta \right)\right|}^{2}=F\left(\Delta \right),\;\Delta \in B\left(R^{3}\right).$$

Let $Y_{lm}\left(\theta ,\varphi \right)$, θ∈[0,π], φ∈[0,2π), l=0,1,⋯, m=−l,⋯,l, be complex spherical harmonics defined by the relation

$$Y_{lm}\left(\theta ,\varphi \right)={\left(-1\right)}^{m}{\left(\frac{(2l+1)(l-m)!}{4\pi (l+m)!}\right)}^{1/2}\exp\left(im\varphi \right)P_{l}^{m}\left(\cos\left(\theta \right)\right),$$

where $P_{l}^{m}\left(\cdot \right)$ are the associated Legendre polynomials with indices *l* and *m*. For spherical harmonics it holds that

$$Y_{l0}\left(0,0\right)=\sqrt{\frac{2l+1}{4\pi }},\;\;\;Y_{l0}\left(\theta ,\varphi \right)=\sqrt{\frac{2l+1}{4\pi }}P_{l0}\left(\cos\theta \right),$$

$$Y_{lm}^{*}\left(\theta ,\varphi \right)={\left(-1\right)}^{m}Y_{l(-m)}\left(\theta ,\varphi \right),$$

$$Y_{lm}\left(\pi -\theta ,\varphi +\pi \right)={\left(-1\right)}^{l}Y_{lm}\left(\theta ,\varphi \right),$$

$$\int_{0}^{\pi }\int_{0}^{2\pi }Y_{lm}^{*}\left(\theta ,\varphi \right)Y_{l^{\prime }m^{\prime }}\left(\theta ,\varphi \right)\sin\theta d\varphi d\theta =\delta_{l}^{l^{\prime }}\delta_{m}^{m^{\prime }},$$

where the symbol * denotes the complex conjugation and $\delta_{l}^{l^{\prime }}$ is the Kronecker delta function. The addition formula for spherical harmonics gives

$$\sum_{m=-l}^{l}Y_{lm}\left(\theta ,\varphi \right)Y_{lm}^{*}\left(\theta ,\varphi \right)=\frac{2l+1}{4\pi }.$$

The Bessel function $J_{\nu }\left(\cdot \right)$ of the first kind of order ν is defined by

$$J_{\nu }\left(\mu \right)=\sum_{n=0}^{\infty }\frac{{\left(-1\right)}^{n}}{n!\Gamma (n+\nu +1)}{\left(\frac{\mu }{2}\right)}^{2n+\nu },$$

where Γ(·) is the Gamma function.

It admits the following representation by the Poisson integral, see (10.9.4) in [27]:

$$J_{\nu }\left(\mu \right)=\frac{2{\left(\mu /2\right)}^{\nu }}{\sqrt{\pi }\Gamma \left(\nu +\frac{1}{2}\right)}\int_{0}^{1}{\left(1-t^{2}\right)}^{\nu -\frac{1}{2}}\cos\left(\mu t\right)dt,\;\nu >-\frac{1}{2}.$$

By the addition theorem for Bessel functions(e.g., [26], p. 14),

(4) $$\eta \left(x\right)=\tilde{\eta }\left(\theta ,\varphi ,r\right)=\pi \sqrt{2}\sum_{l=0}^{\infty }\sum_{m=-l}^{l}Y_{lm}\left(\theta ,\varphi \right)\int_{0}^{\infty }\frac{J_{l+1/2}\left(\mu r\right)}{{\left(\mu r\right)}^{1/2}}\;Z_{lm}\left(d\mu \right),$$

where $Z_{lm}\left(\cdot \right)$ is a family of random measures on $\left(R_{+}^{1},B\left(R_{+}^{1}\right)\right)$, such that

(5) $$EZ_{lm}\left(\Delta_{1}\right)Z_{l^{\prime }m^{\prime }}\left(\Delta_{2}\right)=\delta_{l}^{l^{\prime }}\delta_{m}^{m^{\prime }}G\left(\Delta_{1}\cap \Delta_{2}\right),\;\Delta_{i}\in B\left(R_{+}^{1}\right),\;i=1,2.$$

The stochastic integrals in (3) and (4) are viewed as $L_{2}\left(\Omega \right)$ integrals with the structural measures *F* and *G*, correspondingly.

Let us consider the initial conditions of the form:

(6) $${q\left(x,t\right)|}_{t=0}=\delta \left(x\right),\;{\left.\frac{\partial q(x,t)}{\partial t}\right|}_{t=0}=0,$$

where δ(x) is the Dirac delta function.

Let $Q\left(x,t\right),\;x\in R^{3},\;t\geq 0,$ be the fundamental solution (or the Green’s function) of the initial-value problem (1) and (6), and let

(7) $$H\left(\kappa ,t\right)=\int_{R^{3}}e^{i\langle \kappa ,x\rangle }\;Q\left(x,t\right)\;dx,\;\kappa \in R^{3},\;t\geq 0$$

be its Fourier transform.

The following theorem derives the Fourier transform H(κ,t). Contrary to many models in the literature, for the initial-value problem (1) and (2) it can be explicitly written in terms of elementary functions. Later, this result will be used to obtain the solution q(x,t,ω),$x\in R^{3},\;t\geq 0,\;\omega \in \Omega ,$ and its covariance function.

**Theorem** **1.** *The Fourier transform* (7) *of the initial-value problem* (1) *and* (6) *is given by the formula*

(8) $$\begin{array}{ll} \; & H\left(\kappa ,t\right)=\exp\left(-\frac{c^{2}}{2D}t\right) \end{array}$$

(9) $$\begin{array}{ll} \; & \times \{\left[\cosh\left(ct\sqrt{\frac{c^{2}}{4D^{2}}-{∥\kappa ∥}^{2}}\right)+\frac{c}{2D\sqrt{\frac{c^{2}}{4D^{2}}-{∥\kappa ∥}^{2}}}\sinh\left(ct\sqrt{\frac{c^{2}}{4D^{2}}-{∥\kappa ∥}^{2}}\right)\right]I_{\left\{∥\kappa ∥\leq \frac{c}{2D}\right\}} \end{array}$$

(10) $$\begin{array}{ll} \; & \;+\left[\cos\left(ct\sqrt{{∥\kappa ∥}^{2}-\frac{c^{2}}{4D^{2}}}\right)+\frac{c}{2D\sqrt{{∥\kappa ∥}^{2}-\frac{c^{2}}{4D^{2}}}}\sin\left(ct\sqrt{{∥\kappa ∥}^{2}-\frac{c^{2}}{4D^{2}}}\right)\right]I_{\left\{∥\kappa ∥>\frac{c}{2D}\right\}}\}, \end{array}$$

*where $I_{\left\{\cdot \right\}}$ denotes the indicator function.*

**Proof** **of** **Theorem** **1.** The Fourier transform (7) is the solution of the initial-value problem

(11) $$\begin{array}{c} \;\frac{1}{c^{2}}\frac{d^{2}H\left(\kappa ,t\right)}{dt^{2}}+\frac{1}{D}\frac{dH(\kappa ,t)}{dt}+{∥\kappa ∥}^{2}H\left(\kappa ,t\right)=0,\\ {\;\;H\left(\kappa ,t\right)|}_{t=0}=1,\;{\left.\frac{\partial H(\kappa ,t)}{\partial t}\right|}_{t=0}=0,\;\kappa \in R^{3}. \end{array}$$

The characteristic equation for the ordinary differential equation in (11) is

$$\frac{1}{c^{2}}z^{2}+\frac{1}{D}z+{∥\kappa ∥}^{2}=0,$$

with the roots

(12) $$z_{1}\left(\kappa \right)=-\frac{c^{2}}{2D}-\sqrt{\frac{c^{4}}{4D^{2}}-c^{2}{∥\kappa ∥}^{2}},\;z_{2}\left(\kappa \right)=-\frac{c^{2}}{2D}+\sqrt{\frac{c^{4}}{4D^{2}}-c^{2}{∥\kappa ∥}^{2}}.$$

Therefore, the general solution of the ordinary differential equation in (11) has the form

(13) $$H\left(\kappa ,t\right)=K_{1}\left(\kappa \right)e^{z_{1}\left(\kappa \right)t}+K_{2}\left(\kappa \right)e^{z_{2}\left(\kappa \right)t},$$

where $K_{1}\left(\kappa \right),K_{2}\left(\kappa \right)$ are some functions that do not depend on *t* and $z_{1}\left(\kappa \right),z_{2}\left(\kappa \right)$ are given by (12). From the initial conditions in (11) we obtain the system of equations to find these functions

(14) $$\begin{array}{c} K_{1}\left(\kappa \right)+K_{2}\left(\kappa \right)=1,\;z_{1}\left(\kappa \right)K_{1}\left(\kappa \right)+z_{2}\left(\kappa \right)K_{2}\left(\kappa \right)=0, \end{array}$$

which results in

(15) $$K_{1}\left(\kappa \right)=\frac{1}{2}-\frac{c}{4D\sqrt{\frac{c^{2}}{4D^{2}}-{∥\kappa ∥}^{2}}},\;K_{2}\left(\kappa \right)=\frac{1}{2}+\frac{c}{4D\sqrt{\frac{c^{2}}{4D^{2}}-{∥\kappa ∥}^{2}}}.$$

Thus, by (12) and (13) the solution of the initial-value problem (11) is

$$\begin{array}{ll} H(\kappa ,t) & =\left(\frac{1}{2}-\frac{c}{4D\sqrt{\frac{c^{2}}{4D^{2}}-{∥\kappa ∥}^{2}}}\right)\exp\left[t\left(-\frac{c^{2}}{2D}-\sqrt{\frac{c^{4}}{4D^{2}}-c^{2}{∥\kappa ∥}^{2}}\right)\right]\\ & +\left(\frac{1}{2}+\frac{c}{4D\sqrt{\frac{c^{2}}{4D^{2}}-{∥\kappa ∥}^{2}}}\right)\exp\left[t\left(-\frac{c^{2}}{2D}+\sqrt{\frac{c^{4}}{4D^{2}}-c^{2}{∥\kappa ∥}^{2}}\right)\right]\\ & =\exp\left(-\frac{c^{2}}{2D}t\right)(\frac{1}{2}\left[\exp\left(t\sqrt{\frac{c^{4}}{4D^{2}}-c^{2}{∥\kappa ∥}^{2}}\right)+\exp\left(-t\sqrt{\frac{c^{4}}{4D^{2}}-c^{2}{∥\kappa ∥}^{2}}\right)\right]\\ & +\frac{c}{2D\sqrt{\frac{c^{2}}{4D^{2}}-{∥\kappa ∥}^{2}}}\;\frac{1}{2}\left[\exp\left(t\sqrt{\frac{c^{4}}{4D^{2}}-c^{2}{∥\kappa ∥}^{2}}\right)-\exp\left(-t\sqrt{\frac{c^{4}}{4D^{2}}-c^{2}{∥\kappa ∥}^{2}}\right)\right])\\ & =\exp\left(-\frac{c^{2}}{2D}t\right)\{\cosh\left(t\sqrt{\frac{c^{4}}{4D^{2}}-c^{2}{∥\kappa ∥}^{2}}\right)+\frac{c}{2D\sqrt{\frac{c^{2}}{4D^{2}}-{∥\kappa ∥}^{2}}}\;\\ & \times \sinh\left(t\sqrt{\frac{c^{4}}{4D^{2}}-c^{2}{∥\kappa ∥}^{2}}\right)\}=\exp\left(-\frac{c^{2}}{2D}t\right)\{[\cosh\left(ct\sqrt{\frac{c^{2}}{4D^{2}}-{∥\kappa ∥}^{2}}\right)\\ & +\frac{c}{2D\sqrt{\frac{c^{2}}{4D^{2}}-{∥\kappa ∥}^{2}}}\sinh\left(ct\sqrt{\frac{c^{2}}{4D^{2}}-{∥\kappa ∥}^{2}}\right)]I_{\left\{∥\kappa ∥\leq \frac{c}{2D}\right\}}+[\cos\left(ct\sqrt{{∥\kappa ∥}^{2}-\frac{c^{2}}{4D^{2}}}\right)\\ & +\frac{c}{2D\sqrt{{∥\kappa ∥}^{2}-\frac{c^{2}}{4D^{2}}}}\sin\left(ct\sqrt{{∥\kappa ∥}^{2}-\frac{c^{2}}{4D^{2}}}\right)]I_{\left\{∥\kappa ∥>\frac{c}{2D}\right\}}\}. \end{array}$$

The theorem is proved. □

**Remark** **1.** *The function H(κ,t) given by* (8) *is radial. That is, there exists a function $\tilde{H}\left(\cdot ,\cdot \right)$ defined on (0,∞)×(0,∞) such that $H\left(\kappa ,t\right)=\tilde{H}\left(∥\kappa ∥,t\right)$.*

**Remark** **2.** *c/2D is a cut-off wave number below which the Fourier modes decay exponentially and are non-travelling as in standard heat conduction. At low wave numbers, the governing PDE may be regarded as a delayed diffusion equation, as in Cattaneo’s theory of heat propagation* [17]. *At higher wave numbers, it can easily be seen from the one-dimensional solutions that the Fourier components may be viewed as travelling waves but with exponentially decaying amplitude. At high wave numbers, the governing PDE may be regarded as a damped wave equation.*

Let us denote $\tilde{H}\left(\mu ,t\right)={\tilde{H}}_{1}\left(\mu ,t\right)+{\tilde{H}}_{2}\left(\mu ,t\right),$ such that

(16) $$\begin{array}{cc} {\tilde{H}}_{1}\left(\mu ,t\right) & =\exp\left(-\frac{c^{2}}{2D}t\right)[\cosh\left(ct\sqrt{\frac{c^{2}}{4D^{2}}-\mu^{2}}\right)\\ \; & +\frac{c}{2D\sqrt{\frac{c^{2}}{4D^{2}}-\mu^{2}}}\sinh\left(ct\sqrt{\frac{c^{2}}{4D^{2}}-\mu^{2}}\right)]I_{\left\{|\mu |\leq \frac{c}{2D}\right\}}, \end{array}$$

(17) $$\begin{array}{cc} {\tilde{H}}_{2}\left(\mu ,t\right) & =\exp\left(-\frac{c^{2}}{2D}t\right)[\cos\left(ct\sqrt{\mu^{2}-\frac{c^{2}}{4D^{2}}}\right)\\ \; & +\frac{c}{2D\sqrt{\mu^{2}-\frac{c^{2}}{4D^{2}}}}\sin\left(ct\sqrt{\mu^{2}-\frac{c^{2}}{4D^{2}}}\right)]I_{\left\{|\mu |>\frac{c}{2D}\right\}}. \end{array}$$

**Lemma** **1.** *It holds that*

(18) $$\begin{array}{c} 0\leq {\tilde{H}}_{1}\left(\mu ,t\right)\leq 1, \end{array}$$

*and*

(19) $$|{\tilde{H}}_{2}\left(\mu ,t\right)|\leq \exp\left(-\frac{c^{2}}{2D}t\right)\left[1+\frac{c^{2}}{2D}t\right].$$

**Proof** **of** **Lemma** **1.** It follows from (13)–(15) that for $\left|\mu \right|\leq \frac{c}{2D}$ it holds

$$\begin{array}{ll} {\tilde{H}}_{1}\left(\mu ,t\right) & =e^{z_{2}\left(\mu \right)t}\left(K_{2}\left(\mu \right)+K_{1}\left(\mu \right)e^{(z_{1}\left(\mu \right)-z_{2}\left(\mu \right))t}\right)=e^{z_{2}\left(\mu \right)t}\left(1+\left(e^{(z_{1}\left(\mu \right)-z_{2}\left(\mu \right))t}-1\right)K_{1}\left(\mu \right)\right)\\ & =e^{z_{2}\left(\mu \right)t}\left(1+\left(e^{-\frac{z_{2}\left(\mu \right)}{K_{1}\left(\mu \right)}t}-1\right)K_{1}\left(\mu \right)\right)=\left(1-K_{1}\left(\mu \right)\right)e^{z_{2}\left(\mu \right)t}+K_{1}\left(\mu \right)e^{\left(z_{2}\left(\mu \right)-\frac{z_{2}\left(\mu \right)}{K_{1}\left(\mu \right)}\right)t}. \end{array}$$

Note that ${\tilde{H}}_{1}\left(\mu ,0\right)=1$ for $\left|\mu \right|\leq \frac{c}{2D}$ and

$$\begin{array}{ll} \frac{\partial {\tilde{H}}_{1}\left(\mu ,t\right)}{\partial t} & =\left(1-K_{1}\left(\mu \right)\right)z_{2}\left(\mu \right)e^{z_{2}\left(\mu \right)t}+K_{1}\left(\mu \right)\left(z_{2}\left(\mu \right)-\frac{z_{2}\left(\mu \right)}{K_{1}\left(\mu \right)}\right)e^{z_{2}\left(\mu \right)-\frac{z_{2}\left(\mu \right)}{K_{1}\left(\mu \right)}t}\\ & =\left(1-K_{1}\left(\mu \right)\right)z_{2}\left(\mu \right)e^{z_{2}\left(\mu \right)t}\left(1-e^{-\frac{z_{2}\left(\mu \right)}{K_{1}\left(\mu \right)}t}\right)\leq 0, \end{array}$$

because $z_{2}\left(\mu \right)\leq 0$ and $K_{1}\left(\mu \right)\leq 0$ if $\left|\mu \right|\leq \frac{c}{2D}$. Thus, ${\tilde{H}}_{1}\left(\mu ,t\right)\leq {\tilde{H}}_{1}\left(\mu ,0\right)=1.$

As $|\frac{\sin(x)}{x}|\leq 1$, one obtains the upper bound (19) from the representation (17) for ${\tilde{H}}_{2}\left(\cdot ,\cdot \right)$. □

The following theorem provides the solution of the initial-value problem and its covariance function in terms of the Fourier transform H(κ,t). As the explicit expression of H(κ,t) in terms of elementary functions is given in Theorem 1, it can be used to obtain an explicit expression for the solution and then easily investigate various properties of q(x,t).

**Theorem** **2.** *The solution $q\left(x,t\right)=q\left(x,t,\omega \right),\;x\in R^{3},\;t\geq 0,\;\omega \in \Omega ,$ of the initial-value problem* (1) and (2) *can be written as the convolution*

(20) $$\begin{array}{c} q\left(x,t\right)=\int_{R^{3}}e^{i(\kappa ,x)}H\left(\kappa ,t\right)Z\left(d\kappa \right). \end{array}$$

*The covariance function of the spatio-temporal random field* (20) *is*

(21) $$Cov\left(q\left(x,t\right),q\left(x^{\prime },t^{\prime }\right)\right)=\int_{R^{3}}e^{\langle \kappa ,x-x^{\prime }\rangle }\;H\left(\kappa ,t\right)\;H\left(\kappa ,t^{\prime }\right)\;F\left(d\kappa \right).$$

**Proof** **of** **Theorem** **2.** Notice that

$$\begin{array}{cc} q\left(x,t\right) & =\int_{R^{3}}\eta \left(y\right)\;Q\left(x-y,t\right)\;dy=\int_{R^{3}}\eta \left(x-z\right)\;Q\left(z,t\right)\;d\;z\;\\ & =\int_{R^{3}}e^{i\langle \kappa ,x\rangle }\left[\int_{R^{3}}e^{i\langle \kappa ,-z\rangle }Q\left(z,t\right)dz\right]Z\left(d\kappa \right)=\int_{R^{3}}e^{i\langle \kappa ,x\rangle }\;H\left(\kappa ,t\right)\;Z\left(d\kappa \right), \end{array}$$

where H(κ,t) is given by (8), assuming that the random initial condition has the spectral measure *F*, such that

(22) $$\int_{R^{3}}{\left|H\left(\kappa ,t\right)\right|}^{2}\;F\left(d\kappa \right)<\infty .$$

Under the condition (22), the stochastic integral (20) exists in the $L_{2}\left(\Omega \right)$-sense.

By Lemma 1 the function |H(κ,t)| can be bounded by a constant C(t) which depends only on *t*. Noting that $\int_{R^{3}}{\left|H\left(\kappa ,t\right)\right|}^{2}F\left(d\kappa \right)\leq C\left(t\right)B\left(0\right)$ we obtain (22). The representation (21) immediately follows from (20) and the orthogonality of Z(·). □

## 3. Spherical Random Hyperbolic Diffusion

In this section we investigate a restriction of the spatial-temporal hyperbolic diffusion field from Section 2 to the unit sphere.

Consider the sphere $S^{2}=\{x\in R^{3}:∥x∥=1\}$ in the three-dimensional Euclidean space $R^{3}$ with the Lebesgue measure

$$\tilde{\sigma }\left(dx\right)=\sigma \left(d\theta ,d\varphi \right)=\sin\theta \;d\theta \;d\varphi ,\;\theta \in \left[0,\pi \right],\;\varphi \in \left[0,2\pi \right).$$

A spatio-temporal spherical random field defined on a probability space (Ω,F,P) is a stochastic function

$$T\left(x,t\right)=T\left(x,t,\omega \right)=\tilde{T}\left(\theta ,\varphi ,t\right),\;x\in S^{2},\;t\geq 0.$$

We consider a real-valued spatio-temporal spherical random field *T* with zero mean and finite second-order moments and being continuous in the mean-square sense (see, e.g., Marinucci and Peccati [3] for definitions and other details). Under these conditions, the zero-mean random field *T* can be expanded in the mean-square sense as the Laplace series [25]:

$$\tilde{T}\left(\theta ,\varphi ,t\right)=\sum_{l=0}^{\infty }\sum_{m=-l}^{l}Y_{lm}\left(\theta ,\varphi \right)\;a_{lm}\left(t\right),$$

where the functions $Y_{lm}\left(\theta ,\varphi \right)$ represent the spherical harmonics and the coefficients $a_{lm}\left(t\right)$ are given by the formula

$$a_{lm}\left(t\right)=\int_{0}^{\pi }\int_{0}^{2\pi }\tilde{T}\left(\theta ,\varphi ,t\right)\;Y_{lm}^{*}\left(\theta ,\varphi \right)\;\sin\theta \;d\theta \;d\varphi .$$

We assume that the field *T* is isotropic (in the weak sense), that is, $ET^{2}\left(x,t\right)<\infty$, and $ET\left(x,t\right)T\left(y,t^{\prime }\right)=ET\left(gx,t\right)T\left(gy,t^{\prime }\right)$ for every g∈SO(3), the group of rotations in $R^{3}$. This is equivalent to the condition that the covariance function $E\tilde{T}\left(\theta ,\varphi ,t\right)\tilde{T}\left(\theta^{\prime },\varphi^{\prime },t^{\prime }\right)$ depends only on the angular distance $\gamma =\gamma_{PQ}$ between the points P=(θ,φ) and $Q=(\theta^{\prime },\varphi^{\prime })$ on $S^{2}$ for every $t,t^{\prime }\geq 0$.

The field is isotropic if and only if

(23) $$\begin{array}{c} Ea_{lm}\left(t\right)a_{l^{\prime }m^{\prime }}\left(t^{\prime }\right)=\delta_{l}^{l^{\prime }}\delta_{m}^{m^{\prime }}C_{l}\left(t,t^{\prime }\right),\;-l\leq m\leq l,\;-l^{\prime }\leq m^{\prime }\leq l^{\prime }. \end{array}$$

Hence,

$$Ea_{lm}\left(t\right)a_{lm}\left(t^{\prime }\right)=C_{l}\left(t,t^{\prime }\right),\;m=0,\pm 1,\cdots ,\pm l.$$

The functional series $\left\{C_{l}\left(t,t^{\prime }\right),\;l=0,1,\cdots \right\}$ is called the angular time-dependent power spectrum of the isotropic random field $\tilde{T}\left(\theta ,\varphi ,t\right)$.

We can define a covariance function between two locations with the angular distance γ at times *t* and $t^{\prime }$ by

(24) $$R\left(\cos\gamma ,t,t^{\prime }\right)=ET\left(\theta ,\varphi ,t\right)T\left(\theta^{\prime },\varphi^{\prime },t^{\prime }\right)=\frac{1}{4\pi }\sum_{l=0}^{\infty }\left(2l+1\right)\;C_{l}\left(t,t^{\prime }\right)\;P_{l}\left(\cos\gamma \right),$$

where $P_{l}\left(x\right)=\frac{1}{2^{l}\;l!}\;\frac{d^{l}}{dx^{l}}{\left(x^{2}-1\right)}^{l}$ is the *l*-th Legendre polynomial.

If $\tilde{T}\left(\theta ,\varphi ,t\right)$ is a zero-mean isotropic Gaussian field, then the coefficients $a_{lm}\left(t\right),\;m=-l,\cdots ,l,$l≥ 1, are complex-valued Gaussian stochastic processes with

$$Ea_{lm}\left(t\right)=0,\;Ea_{lm}\left(t\right)a_{l^{\prime }m^{\prime }}\left(t^{\prime }\right)=\delta_{l}^{l^{\prime }}\delta_{m}^{m^{\prime }}C_{l}\left(t,t^{\prime }\right).$$

By Remark 1, the random field $q\left(x,t\right),\;x\in R^{3}$ given by (20) is homogeneous and isotropic in x, and hence its covariance function (21) can be written in the form:

$$\begin{array}{ll} \mathrm{Cov}\left(q\left(x,t\right),q\left(x^{\prime },t^{\prime }\right)\right)= & \int_{0}^{\infty }\frac{\sin(\mu ∥x-x^{\prime }∥)}{\mu ∥x-x^{\prime }∥}\;\tilde{H}\left(\mu ,t\right)\;\tilde{H}\left(\mu ,t^{\prime }\right)\;G\left(d\mu \right)\\ & =2\pi^{2}\sum_{l=0}^{\infty }\sum_{m=-l}^{l}Y_{lm}\left(\theta ,\varphi \right)\;Y_{lm}^{*}\left(\theta^{\prime },\varphi^{\prime }\right)\\ & \times \int_{0}^{\infty }\frac{J_{l+1/2}\left(\mu r\right)}{{\left(\mu r\right)}^{1/2}}\;\frac{J_{l+1/2}\left(\mu r^{\prime }\right)}{{\left(\mu r^{\prime }\right)}^{1/2}}\;\tilde{H}\left(\mu ,t\right)\;\tilde{H}\left(\mu ,t^{\prime }\right)\;G\left(d\mu \right), \end{array}$$

where (r,θ,φ) and $\left(r^{\prime },\theta^{\prime },\varphi^{\prime }\right)$ are spherical coordinates of x and $x^{\prime }$ respectively.

Using the Karhunen theorem we obtain the following spectral representation of the random field:

(25) $$q\left(x,t\right)=\tilde{q}\left(r,\theta ,\varphi ,t\right)=\pi \sqrt{2}\sum_{l=0}^{\infty }\sum_{m=-l}^{l}Y_{lm}\left(\theta ,\varphi \right)\int_{0}^{\infty }\frac{J_{l+1/2}\left(r\mu \right)}{{\left(r\mu \right)}^{1/2}}\;\tilde{H}\left(\mu ,t\right)\;Z_{lm}\left(d\mu \right),$$

where the random measures $Z_{lm}\left(\cdot \right)$ are given in (5).

Similarly to the condition (22) the isotropic measure G(·) satisfies the following condition if the field has a finite variance:

$$\int_{0}^{\infty }\mu^{2}\;{\left|\tilde{H}\left(\mu ,t\right)\right|}^{2}\;G\left(d\mu \right)<\infty .$$

Subclasses of covariance functions of the isotropic fields on the sphere can be obtained from covariance functions of homogeneous isotropic random fields in Euclidean space, since a restriction of the homogeneous and isotropic random field to the sphere yields an isotropic spherical field (e.g., [25], p. 76).

Consider two locations x and $x^{\prime }$ on the unit sphere $S^{2}$ with the angle γ∈[0,π] between them. Then, the Euclidean distance between these two points is $2\sin\frac{\gamma }{2}$, the inner product is $\langle x,x^{\prime }\rangle =\cos\gamma$, which gives a direct correspondence between the covariance function $R_{0}(∥x-x^{\prime }∥,t,t^{\prime })$ in the Euclidean space and the covariance function $R\left(\cos\gamma ,t,t^{\prime }\right)=R_{0}\left(2\sin\frac{\gamma }{2},t,t^{\prime }\right)$ on the sphere for every fixed $t,t^{\prime }\geq 0$. Thus, the restriction of the homogeneous and isotropic hyperbolic diffusion field (25) to $S^{2}$ is an isotropic spherical random field for every fixed $t,t^{\prime }\geq 0$. We will call it the spherical hyperbolic diffusion isotropic random field $T_{H}\left(x,t\right),\;x\in S^{2},\;t\geq 0$.

Its covariance function is of the form:

(26) $$\mathrm{Cov}\left(T_{H}\left(x,t\right),T_{H}\left(x^{\prime },t^{\prime }\right)\right)=R\left(\cos\gamma ,t,t^{\prime }\right)=\int_{0}^{\infty }\frac{\sin(2\mu \sin\frac{\gamma }{2})}{2\mu \sin\frac{\gamma }{2}}\;\tilde{H}\left(\mu ,t\right)\;\tilde{H}\left(\mu ,t^{\prime }\right)\;G\left(d\mu \right).$$

By the addition theorem for Bessel functions, the random field $T_{H}\left(x,t\right)={\tilde{T}}_{H}\left(\theta ,\varphi ,t\right)$ has the following spectral representation:

(27) $${\tilde{T}}_{H}\left(\theta ,\varphi ,t\right)=\sum_{l=0}^{\infty }\sum_{m=-l}^{l}Y_{lm}\left(\theta ,\varphi \right)\;a_{lm}\left(t\right),$$

where

(28) $$a_{lm}\left(t\right)=\pi \sqrt{2}\int_{0}^{\infty }\frac{J_{l+1/2}\left(\mu \right)}{\sqrt{\mu }}\;\tilde{H}\left(\mu ,t\right)\;Z_{lm}\left(d\mu \right)$$

and the random measure $Z_{lm}\left(\cdot \right)$ satisfies (5).

Thus, the angular spectrum of the isotropic spherical random field $T_{H}\left(x,t\right)$ is given by the formula

(29) $$C_{l}\left(t,t^{\prime }\right)=2\pi^{2}\int_{0}^{\infty }\frac{J_{l+1/2}^{2}\left(\mu \right)}{\mu }\;\tilde{H}\left(\mu ,t\right)\;\tilde{H}\left(\mu ,t^{\prime }\right)\;G\left(d\mu \right).$$

Therefore, we obtained the following result.

**Theorem** **3.** *Consider the random initial-value problem* (1) *and* (2)*, in which $\eta \left(x\right),\;x\in R^{3}$, is a homogeneous isotropic random field with the isotropic spectral measure G(·) given by* (5).

*Then, the restriction of the spatio-temporal hyperbolic-diffusion random field* (25) *to the sphere $S^{2}$ is an isotropic spatio-temporal spherical random field with the following angular spectrum:*

$$\begin{array}{cc} C_{l}\left(t,t^{\prime }\right)=2\pi^{2} & [\int_{0}^{\frac{c}{2D}}\frac{J_{l+1/2}^{2}\left(\mu \right)}{\mu }\;{\tilde{H}}_{1}\left(\mu ,t\right)\;{\tilde{H}}_{1}\left(\mu ,t^{\prime }\right)\;G\left(d\mu \right)\\ & +\int_{\frac{c}{2D}}^{\infty }\frac{J_{l+1/2}^{2}\left(\mu \right)}{\mu }\;{\tilde{H}}_{2}\left(\mu ,t\right)\;{\tilde{H}}_{2}\left(\mu ,t^{\prime }\right)\;G\left(d\mu \right)]. \end{array}$$

*The field and its covariance functions are given by* (27) *and* (26)*, respectively.*

This result investigates the restriction of the spatio-temporal hyperbolic-diffusion random field to the sphere $S^{2}.$ It shows how the angular power spectrum $C_{l}\left(t,t^{\prime }\right)$ of the restriction depends on the Fourier transform $\tilde{H}\left(\mu ,t\right).$ Hence, one can explicitly compute coefficients $C_{l}\left(t,t^{\prime }\right)$ and study the contributions of different spherical harmonics to the spatial-temporal field $T_{H}\left(x,t\right).$

Notice that $T_{H}\left(x,0\right)=\eta \left(x\right),\;x\in S^{2}$. The angular power spectrum of η(x), $x\in S^{2}$, will be denoted by $C_{l}$, l=0,1,⋯ For spherical random fields with finite variances, it holds

(30) $$\begin{array}{c} \sum_{l=0}^{\infty }\left(2l+1\right)C_{l}<\infty . \end{array}$$

**Lemma** **2.** *If* (30) *holds true, then*

$$\sum_{l=0}^{\infty }\left(2l+1\right)C_{l}\left(t,t^{\prime }\right)<\infty .$$

**Proof** **of** **Lemma** **2.** By Theorem 3

(31) $$\begin{array}{ll} \sum_{l=0}^{\infty }\left(2l+1\right)C_{l}\left(t,t^{\prime }\right) & =2\pi^{2}\sum_{l=0}^{\infty }\left(2l+1\right)\int_{0}^{c/2D}\frac{J_{l+\frac{1}{2}}^{2}\left(\mu \right)}{\mu }{\tilde{H}}_{1}\left(\mu ,t\right){\tilde{H}}_{1}\left(\mu ,t^{\prime }\right)G\left(d\mu \right)\\ & +2\pi^{2}\sum_{l=0}^{\infty }\left(2l+1\right)\int_{c/2D}^{\infty }\frac{J_{l+\frac{1}{2}}^{2}\left(\mu \right)}{\mu }{\tilde{H}}_{2}\left(\mu ,t\right){\tilde{H}}_{2}\left(\mu ,t^{\prime }\right)G\left(d\mu \right)\\ & \leq 2\pi^{2}\cdot \underset{\mu <\frac{c}{2D}}{\sup}|{\tilde{H}}_{1}\left(\mu ,t\right){\tilde{H}}_{1}\left(\mu ,t^{\prime }\right)|\cdot \sum_{l=0}^{\infty }\left(2l+1\right)\int_{0}^{c/2D}J_{l+\frac{1}{2}}^{2}\left(\mu \right)G\left(d\mu \right)\\ & +2\pi^{2}\cdot \underset{\mu \geq \frac{c}{2D}}{\sup}|{\tilde{H}}_{2}\left(\mu ,t\right){\tilde{H}}_{2}\left(\mu ,t^{\prime }\right)|\cdot \sum_{l=0}^{\infty }\left(2l+1\right)\int_{c/2D}^{\infty }\frac{J_{l+\frac{1}{2}}^{2}\left(\mu \right)}{\mu }G\left(d\mu \right). \end{array}$$

Now, combining (31) and Lemma 1, one gets

(32) $$\begin{array}{ll} \sum_{l=0}^{\infty }\left(2l+1\right)C_{l}\left(t,t^{\prime }\right) & \leq 2\pi^{2}\int_{0}^{c/2D}\sum_{l=0}^{\infty }\left(2l+1\right)\frac{J_{l+\frac{1}{2}}^{2}\left(\mu \right)}{\mu }G\left(d\mu \right)+\exp\left(-\frac{c^{2}}{D}t\right){\left(1+\frac{c^{2}}{2D}t\right)}^{2}\\ & \times 2\pi^{2}\int_{c/2D}^{\infty }\sum_{l=0}^{\infty }\left(2l+1\right)\frac{J_{l+\frac{1}{2}}^{2}\left(\mu \right)}{\mu }G\left(d\mu \right)\leq \sum_{l=0}^{\infty }\left(2l+1\right)C_{l}, \end{array}$$

as $\sup_{x\geq 0}\left(x+1\right)e^{-x}=1$, ${\tilde{H}}_{1}\left(\mu ,0\right)={\tilde{H}}_{2}\left(\mu ,0\right)=1$, and $C_{l}\left(0,0\right)=C_{l}$. This completes the proof. □

**Remark** **3.** *It follows from Lemma* 2 *and the estimate $|P_{l}\left(\cos\theta \right)|\leq 1$ that the solution’s covariance function given by* (24) *is finite if the initial condition $\eta \left(x\right),\;x\in S^{2}$, has a finite variance.*

## 4. Smoothness of Solutions

Numerous problems in mathematical physics and geosciences require studying the regularity properties of solutions of differential equations. Smoothness, boundedness of derivatives or Hölder continuity conditions are often used to describe and investigate local changes and growth rates of solutions. Knowing regularity properties is also essential for an adequate approximation of SPDE solutions. In those cases where solutions are given by infinite series, it is a rather difficult mathematical problem, as the tail terms of such series can accumulate.

In this section, we investigate the Hölder-type continuity of the solution $\tilde{T}\left(\theta ,\varphi ,t\right)$ given by (27) on the sphere. Estimations of the closeness of $\tilde{T}$ values at spherical points (θ,φ) and $\left(\theta^{\prime },\varphi^{\prime }\right)$ are obtained. We demonstrate how they depend on the decay of the angular power spectrum and provide some specifications in terms of the spectral measure G(·).

First, we obtain the continuity of the solution with respect to the geodesic distance on the sphere. To prove this we use the approach from Corollary 5 in [8].

**Theorem** **4.** *Let ${\tilde{T}}_{H}\left(\theta ,\varphi ,t\right)$ be the solution of the initial value problem*(1)*–* (2) *and the random initial condition η(x), $x\in S^{2}$, has the angular power spectrum $\left\{C_{l},l=0,1,2,\cdots \right\}$ satisfying the assumption*

(33) $$\begin{array}{c} \sum_{l=0}^{\infty }{\left(2l+1\right)}^{1+2\alpha }C_{l}<\infty ,\;\alpha \in \left(0,1\right]. \end{array}$$

(a) *Then, for t>0*
  $$MSE\left({\tilde{T}}_{H}\left(\theta ,\varphi ,t\right)-{\tilde{T}}_{H}\left(\theta^{\prime },\varphi^{\prime },t\right)\right)\leq C\sum_{l=0}^{\infty }{\left(2l+1\right)}^{1+2\alpha }C_{l}{\left(1-\cos\gamma \right)}^{\alpha },$$
  *where γ is the angle between directions (θ,φ) and $\left(\theta^{\prime },\varphi^{\prime }\right)$.*
(b) *If the measure G(·) has its support in $\left[\frac{c}{2D},\infty \right)$, then*
  $$MSE\left({\tilde{T}}_{H}\left(\theta ,\varphi ,t\right)-{\tilde{T}}_{H}\left(\theta^{\prime },\varphi^{\prime },t\right)\right)\leq C\exp\left(-\frac{c^{2}}{D}t\right){\left(1+\frac{c^{2}}{2D}t\right)}^{2}\sum_{l=0}^{\infty }{\left(2l+1\right)}^{1+2\alpha }C_{l}{\left(1-\cos\gamma \right)}^{\alpha }.$$

**Proof** **of** **Theorem** **4.** (a) It follows from (23), (24), (27) and (32) that

$$\begin{array}{ll} MSE\left({\tilde{T}}_{H}\left(\theta ,\varphi ,t\right)-{\tilde{T}}_{H}\left(\theta^{\prime },\varphi^{\prime },t\right)\right) & =2Var\left({\tilde{T}}_{H}\left(\theta ,\varphi ,t\right)\right)-2\mathrm{Cov}\left({\tilde{T}}_{H}\left(\theta ,\varphi ,t\right){\tilde{T}}_{H}\left(\theta^{\prime },\varphi^{\prime },t\right)\right)\\ & =\frac{1}{2\pi }\sum_{l=0}^{\infty }\left(2l+1\right)C_{l}\left(t,t\right)\left(1-P_{l}\left(\cos\gamma \right)\right)\\ & \leq \frac{1}{2\pi }\sum_{l=0}^{\infty }\left(2l+1\right)C_{l}\left(1-P_{l}\left(\cos\gamma \right)\right). \end{array}$$

Applying the property of Legendre polynomials, [23], p. 16,

$$|1-P_{l}{\left(\cos\gamma \right)|\leq 2\left(1-\cos\gamma \right)}^{\alpha }{\left(l\left(l+1\right)\right)}^{\alpha },\;\alpha \in \left(0,1\right],$$

one obtains the statement (a) of the theorem.

(b) It follows from the proof of (32) that in the case of $G(\left[0,\frac{c}{2D}\right])=0$ it holds

$$C_{l}\left(t,t\right)\leq \exp\left(-\frac{c^{2}}{D}t\right){\left(1+\frac{c^{2}}{2D}t\right)}^{2}C_{l}.$$

The remaining steps are similar to the proof in (a). □

When the geodesic distance γ vanishes (i.e., γ→0), it is easy to see that ${\left(1-\cos\gamma \right)}^{\alpha }\to 0$ and, therefore, $MSE\left({\tilde{T}}_{H}\left(\theta ,\varphi ,t\right)-{\tilde{T}}_{H}\left(\theta^{\prime },\varphi^{\prime },t\right)\right)\to 0$ as well.

The next two results provide conditions on the field’s spectrum that guarantee the Hölder-type regularity of ${\tilde{T}}_{H}\left(\theta ,\varphi ,t\right).$

**Theorem** **5.** *If the measure G(·) has a bounded support [0,δ],δ>0, then*

(34) $$\begin{array}{c} MSE\left({\tilde{T}}_{H}\left(\theta ,\varphi ,t\right)-{\tilde{T}}_{H}\left(\theta^{\prime },\varphi^{\prime },t\right)\right)\leq C\left(1-\cos\gamma \right),\;\mathrm{when}\;\gamma \to 0+, \end{array}$$

*even for the case of α=0 in* (33).

**Proof** **of** **Theorem** **5.** Indeed, by (26) we get

$$\begin{array}{cc} MSE\left({\tilde{T}}_{H}\left(\theta ,\varphi ,t\right)-{\tilde{T}}_{H}\left(\theta^{\prime },\varphi^{\prime },t\right)\right) & =2\int_{0}^{\infty }\left(1-\frac{\sin(2\mu \sin\frac{\gamma }{2})}{2\mu \sin\frac{\gamma }{2}}\right)\;H^{2}\left(\mu ,t\right)\;G\left(d\mu \right)\\ \; & =2\int_{0}^{\delta }\left(1-\frac{\sin(2\mu \sin\frac{\gamma }{2})}{2\mu \sin\frac{\gamma }{2}}\right)\;H^{2}\left(\mu ,t\right)\;G\left(d\mu \right). \end{array}$$

For μ∈[0,δ] it holds $2\mu \sin\frac{\gamma }{2}\to 0$, when $\gamma \to 0_{+}$, and therefore

$$\begin{array}{c} |1-\frac{\sin(2\mu \sin\frac{\gamma }{2})}{2\mu \sin\frac{\gamma }{2}}|=|\sum_{k=1}^{\infty }\frac{{\left(-1\right)}^{k}}{(2k+1)!}{\left(2\mu \sin\frac{\gamma }{2}\right)}^{2k+1}|\leq \frac{{\left(2\mu \sin\frac{\gamma }{2}\right)}^{2}}{3!}. \end{array}$$

Hence,

$$MSE\left({\tilde{T}}_{H}\left(\theta ,\varphi ,t\right)-{\tilde{T}}_{H}\left(\theta^{\prime },\varphi^{\prime },t\right)\right)\leq C\sin^{2}\frac{\gamma }{2}\int_{0}^{\delta }\mu^{2}H^{2}\left(\mu ,t\right)G\left(d\mu \right)$$

and (34) follows from Lemma 1. □

The next result gives sufficient conditions to guarantee (33).

**Theorem** **6.** *Suppose that $\int_{0}^{\infty }e^{\mu^{2}/4}G\left(d\mu \right)<\infty$. Then,* (33) *holds true.*

**Proof** **of** **Theorem** **6.** By the Poisson integral representation of the Bessel function it follows that

$$\begin{array}{cc} \sum_{l=0}^{\infty }{\left(2l+1\right)}^{1+2\alpha }C_{l} & =2\pi^{2}\int_{0}^{\infty }\sum_{l=0}^{\infty }{\left(2l+1\right)}^{1+2\alpha }J_{l+\frac{1}{2}}^{2}\left(\mu \right)\frac{G(d\mu )}{\mu }\\ \; & \leq C\int_{0}^{\infty }\sum_{l=0}^{\infty }{\left(2l+1\right)}^{1+2\alpha }\frac{\mu^{2l+1}}{2^{2l+1}\Gamma^{2}\left(l+1\right)}\frac{G(d\mu )}{\mu }\\ \; & \leq C\int_{0}^{\infty }\mu \sum_{l=0}^{\infty }\frac{{\left(\mu^{2}/4\right)}^{l}}{l!}\frac{{\left(2l+1\right)}^{1+2\alpha }}{l!}\frac{G(d\mu )}{\mu }\leq C\int_{0}^{\infty }e^{\frac{\mu^{2}}{4}}G\left(d\mu \right), \end{array}$$

as 1+2α≤3. □

## 5. Short and Long Memory

Investigating statistical dependence between measurements at two points with increasing time or spatial distance between them is an important issue for practical temporal or spatial predictions. The spatial domain of the considered random fields is restricted to the sphere $S^{2}$ with the geodesic distance γ. Note that this distance is bounded to the interval [0,π], but time *t* can unboundedly increase and takes values in [0,+∞). Hence, this section investigates only temporal statistical dependencies, namely, slow or fast decays of covariance functions in time. The corresponding cases represent long or short memory scenarios.

In this section we use the representation (26) of covariance functions to investigate the structure of dependences of $T_{H}\left(x,t\right)$ over time. We demonstrate that conditional on the spectral isotropic measure G(·) of the initial random condition $\eta \left(x\right),\;x\in R^{3}$, the random field $T_{H}\left(x,t\right)$ can exhibit short- or long-range dependence.

The random field $T_{H}\left(x,t\right)$ will be called short-range dependent if

(35) $$\int_{0}^{+\infty }\left|R\left(\cos\gamma ,t+h,t\right)\right|dh<+\infty ,$$

for all t≥0 and γ∈[0,π]. If the integral in (35) is divergent, the field is called long-range dependent.

Results that link behaviours of covariance functions at infinity and spectral measures at the origin are called Abelian–Tauberian theorems. A very detailed overview of such results for random fields can be found in [28].

First we investigate the case of $x=x^{\prime }$ in (26) (i.e., the behaviour of R(1,t+h,t)).

**Theorem** **7.** *For $x=x^{\prime }$ the random field $T_{H}\left(x,t\right)$ exhibits short-range dependence if and only if $\mu^{-2}G\left(d\mu \right)$ is integrable in a neighbourhood of zero.*

**Proof** **of** **Theorem** **7.** It follows from (16), (17) and (26) that

$$\int_{0}^{+\infty }\left|R\left(1,t+h,t\right)\right|dh=\int_{0}^{+\infty }|\int_{0}^{c/2D}{\tilde{H}}_{1}\left(\mu ,t+h\right){\tilde{H}}_{1}\left(\mu ,t\right)G\left(d\mu \right)$$

$$+\int_{c/2D}^{+\infty }{\tilde{H}}_{2}\left(\mu ,t+h\right){\tilde{H}}_{2}\left(\mu ,t\right)G\left(d\mu \right)|dh.$$

Using the upper bound from (19) we get

$$\int_{0}^{+\infty }|\int_{c/2D}^{+\infty }{\tilde{H}}_{2}\left(\mu ,t+h\right){\tilde{H}}_{2}\left(\mu ,t\right)G\left(d\mu \right)|dh\leq \exp\left(-\frac{c^{2}}{2D}t\right)\left[1+\frac{c^{2}}{2D}t\right]\cdot G\left(\left[\frac{c}{2D},+\infty \right)\right)$$

(36) $$\times \int_{0}^{+\infty }\exp\left(-\frac{c^{2}}{2D}h\right)\left[1+\frac{c^{2}}{2D}\left(t+h\right)\right]dh<+\infty .$$

Hence, to study the integrability of the covariance function |R(1,t+h,t)| one has to investigate the integral

(37) $$\int_{0}^{+\infty }|\int_{0}^{c/2D}{\tilde{H}}_{1}\left(\mu ,t+h\right){\tilde{H}}_{1}\left(\mu ,t\right)G\left(d\mu \right)|dh.$$

As ${\tilde{H}}_{1}\left(\mu ,t\right)>0$ for $\left|\mu \right|\leq \frac{c}{2D}$, t≥0, it is equivalent to studying the integral

$$\int_{0}^{c/2D}\int_{0}^{+\infty }{\tilde{H}}_{1}\left(\mu ,t+h\right){\tilde{H}}_{1}\left(\mu ,t\right)dh\;G\left(d\mu \right),$$

or, by (16) and $\cosh\left(\frac{c^{2}t}{2D}\sqrt{1-\frac{4D^{2}}{c^{2}}\mu^{2}}\right)\in \left[1,\cosh\frac{c^{2}t}{2D}\right]$ for $\mu \in \left[0,\frac{c}{2D}\right]$, to investigating the finiteness of the integral

$$\int_{0}^{c/2D}\int_{0}^{+\infty }\left(\exp\left(-\frac{c^{2}}{2D}h\left(1-\sqrt{1-\frac{4D^{2}}{c^{2}}\mu^{2}}\right)\right)\left[1+\frac{1}{\sqrt{1-\frac{4D^{2}}{c^{2}}\mu^{2}}}\right]\right.$$

$$\begin{array}{cc} \; & \left.-\exp\left(-\frac{c^{2}}{2D}h\left(1+\sqrt{1-\frac{4D^{2}}{c^{2}}\mu^{2}}\right)\right)\frac{1}{\sqrt{1-\frac{4D^{2}}{c^{2}}\mu^{2}}}\right)\left(1+\frac{\sinh\left(\frac{c^{2}t}{2D}\sqrt{1-\frac{4D^{2}}{c^{2}}\mu^{2}}\right)}{\sqrt{1-\frac{4D^{2}}{c^{2}}\mu^{2}}}\right)dh\;G\left(d\mu \right)\\ \; & =\frac{2D}{c^{2}}\int_{0}^{c/2D}\left(\frac{1}{1-\sqrt{1-\frac{4D^{2}}{c^{2}}\mu^{2}}}+\left(\frac{1}{1-\sqrt{1-\frac{4D^{2}}{c^{2}}\mu^{2}}}-\frac{1}{1+\sqrt{1-\frac{4D^{2}}{c^{2}}\mu^{2}}}\right)\frac{1}{\sqrt{1-\frac{4D^{2}}{c^{2}}\mu^{2}}}\right) \end{array}$$

$$\begin{array}{cc} \; & \times \left(1+\frac{\sinh\left(\frac{c^{2}t}{2D}\sqrt{1-\frac{4D^{2}}{c^{2}}\mu^{2}}\right)}{\sqrt{1-\frac{4D^{2}}{c^{2}}\mu^{2}}}\right)G\left(d\mu \right). \end{array}$$

Noting that $\frac{\sin(h)}{h}\in \left[0,\frac{\sinh(A)}{A}\right]$ on [0,A], A>0, we obtain that (37) is finite if and only if the following integral converges:

$$\int_{0}^{c/2D}\left(\frac{1}{1-\sqrt{1-\frac{4D^{2}}{c^{2}}\mu^{2}}}+\frac{c^{2}}{2D^{2}\mu^{2}}\right)G\left(d\mu \right)=\frac{c^{2}}{4D^{2}}\int_{0}^{c/2D}\frac{3+\sqrt{1-\frac{4D^{2}}{c^{2}}\mu^{2}}}{\mu^{2}}G\left(d\mu \right).$$

The last integral is finite only if $\int_{0}^{\varepsilon }\frac{G(d\mu )}{\mu^{2}}<\infty ,\;\varepsilon >0$, which completes the proof. □

Now we extend Theorem 7 to the case of arbitrary x and $x^{\prime }$ from $S^{2}$.

**Theorem** **8.** *The random field $T_{H}\left(x,t\right)$ is short-range dependent if and only if $\mu^{-2}G\left(d\mu \right)$ is integrable in the neighbourhood of the origin.*

**Proof** **of** **Theorem** **8.** Note that by (26) the integrators in $R(\cos\gamma ,t^{\prime },t)$ and $R(1,t^{\prime },t)$ differ only by a multiplier $\frac{\sin\left(2\mu \sin\frac{\gamma }{2}\right)}{2\mu \sin\frac{\gamma }{2}}$.

Thus,

$$\int_{0}^{+\infty }\left|R\left(\cos\gamma ,t+h,t\right)\right|dh=\int_{0}^{+\infty }|\int_{0}^{c/2D}\frac{\sin\left(2\mu \sin\frac{\gamma }{2}\right)}{2\mu \sin\frac{\gamma }{2}}{\tilde{H}}_{1}\left(\mu ,t+h\right){\tilde{H}}_{1}\left(\mu ,t\right)G\left(d\mu \right)$$

$$+\int_{c/2D}^{+\infty }\frac{\sin\left(2\mu \sin\frac{\gamma }{2}\right)}{2\mu \sin\frac{\gamma }{2}}{\tilde{H}}_{2}\left(\mu ,t+h\right){\tilde{H}}_{2}\left(\mu ,t\right)G\left(d\mu \right)|dh.$$

It follows from the estimates (19), (36) and the inequality $|\frac{\sin(x)}{x}|\leq 1$ that

$$\int_{0}^{+\infty }|\int_{c/2D}^{+\infty }\frac{\sin\left(2\mu \sin\frac{\gamma }{2}\right)}{2\mu \sin\frac{\gamma }{2}}{\tilde{H}}_{2}\left(\mu ,t+h\right){\tilde{H}}_{2}\left(\mu ,t\right)G\left(d\mu \right)|dh<+\infty .$$

Now, note that for γ∈(0,π) the interval $\left[0,c/2D\right)$ can be split into a finite number of subintervals

$$\left[0,c/2D\right)=\overset{K}{\underset{k=1}{⋃}}\left[\frac{\pi }{2\sin\frac{\gamma }{2}}\left(k-1\right),\frac{\pi }{2\sin\frac{\gamma }{2}}k\right)⋃\left[\frac{\pi }{2\sin\frac{\gamma }{2}}K,\frac{c}{2D}\right),$$

where $K=\left[\frac{c\sin\frac{\gamma }{2}}{\pi D}\right]$ and [a] denotes the integer part of *a*. The ratio $\frac{\sin\left(2\mu \sin\frac{\gamma }{2}\right)}{2\mu \sin\frac{\gamma }{2}}$ has the same sign on each of these subintervals. Therefore, similar to the proof of Theorem 7 we obtain the sufficient and necessary condition for the integrability of $\left|R(\cos\gamma ,t^{\prime },t)\right|$

$$\int_{0}^{\frac{\pi }{2\sin\frac{\gamma }{2}}}\frac{\sin\left(2\mu \sin\frac{\gamma }{2}\right)}{2\mu \sin\frac{\gamma }{2}}\frac{G(d\mu )}{\mu^{2}}<\infty .$$

Note that by $\lim_{\mu \to 0}\frac{\sin(\mu )}{\mu }=1$ this condition is equivalent to the one in Theorem 7. This completes the proof. □

## 6. Approximations to Solutions

The results in the previous sections were based on the series representation of the random field ${\tilde{T}}_{H}\left(\theta ,\varphi ,t\right).$ In applications and numerical studies, only a finite number of series terms are available. Hence, one has to investigate the behaviours of finite cumulative sums. This section provides an analysis of truncated series expansions of the solution field ${\tilde{T}}_{H}\left(\theta ,\varphi ,t\right)$ and shows the role of the decay rate of the angular power spectrum. These results can be used to determine the number of terms for a given accuracy of approximate solutions.

This section introduces and studies approximate solutions of the initial value problem (1) and (2). A mean-square convergence rate to the diffusion field in terms of the angular power spectrum $C_{l}$ is obtained. Then, several specifications in terms of the measure G(·) are discussed.

We define the approximation ${\tilde{T}}_{H,L}\left(\theta ,\varphi ,t\right)$ of the truncation degree L∈N to the solution ${\tilde{T}}_{H}\left(\theta ,\varphi ,t\right)$ given by (27) as

$${\tilde{T}}_{H,L}\left(\theta ,\varphi ,t\right)=\sum_{l=0}^{L-1}Y_{lm}\left(\theta ,\varphi \right)\;a_{lm}\left(t\right),\;\theta \in \left[0,\pi \right],\;\varphi \in \left[0,2\pi \right),\;t\geq 0.$$

The next result provides the convergence rate of ${\tilde{T}}_{H,L}\left(\theta ,\varphi ,t\right)$ to ${\tilde{T}}_{H}\left(\theta ,\varphi ,t\right)$ when L→∞.

**Theorem** **9.** *Let ${\tilde{T}}_{H}\left(\theta ,\varphi ,t\right)$ be the solution to the initial value problem*(1) *and* (2) *and ${\tilde{T}}_{H,L}\left(\theta ,\varphi ,t\right)$ the corresponding approximation of truncation degree L∈N. Then,*

$$\begin{array}{c} \underset{t\geq 0}{\sup}{∥{\tilde{T}}_{H}\left(\theta ,\varphi ,t\right)-{\tilde{T}}_{H,L}\left(\theta ,\varphi ,t\right)∥}_{L_{2}\left(\Omega \times S^{2}\right)}\leq \frac{1}{2\sqrt{\pi }}{\left(\sum_{l=L}^{\infty }\left(2l+1\right)C_{l}\right)}^{1/2}. \end{array}$$

**Proof** **of** **Theorem** **9.** Note that by properties of $a_{lm}\left(t\right)$ we get

$$E({\tilde{T}}_{H}\left(\theta ,\varphi ,t\right)-{\tilde{T}}_{H,L}\left(\theta ,\varphi ,t\right))=0$$

for all L∈N, θ∈[0,π], φ∈[0,2π) and t≥0.

Then, by (23) and (27) it follows that

$$\begin{array}{cc} ∥{\tilde{T}}_{H}\left(\theta ,\varphi ,t\right)-{\tilde{T}}_{H,L}{\left(\theta ,\varphi ,t\right)∥}_{L_{2}\left(\Omega \times S^{2}\right)} & ={\left(\sum_{l=L}^{\infty }\sum_{m=-l}^{l}Y_{lm}\left(\theta ,\varphi \right)Y_{lm}^{*}\left(\theta ,\varphi \right)E\left(a_{lm}\left(t\right)a_{lm}^{*}\left(t\right)\right)\right)}^{1/2}\\ \; & ={\left(\sum_{l=L}^{\infty }\sum_{m=-l}^{l}Y_{lm}\left(\theta ,\varphi \right)Y_{lm}^{*}\left(\theta ,\varphi \right)C_{l}\left(t,t\right)\right)}^{1/2}. \end{array}$$

Using the addition formula for spherical harmonics one gets

(38) $$\begin{array}{c} ∥{\tilde{T}}_{H}\left(\theta ,\varphi ,t\right)-{\tilde{T}}_{H,L}{\left(\theta ,\varphi ,t\right)∥}_{L_{2}\left(\Omega \times S^{2}\right)}=\frac{1}{2\sqrt{\pi }}{\left(\sum_{l=L}^{\infty }\left(2l+1\right)C_{l}\left(t,t\right)\right)}^{1/2}. \end{array}$$

Finally, by (32)

$$\begin{array}{c} ∥{\tilde{T}}_{H}\left(\theta ,\varphi ,t\right)-{\tilde{T}}_{H,L}{\left(\theta ,\varphi ,t\right)∥}_{L_{2}\left(\Omega \times S^{2}\right)}\leq \frac{1}{2\sqrt{\pi }}{\left(\sum_{l=L}^{\infty }\left(2l+1\right)C_{l}\right)}^{1/2}. \end{array}$$

 □

For the SPDE model studied in [8] it was shown that its solution has an exponential decay in *t* and the corresponding approximation error can be bounded as

(39) $$\begin{array}{c} ∥u\left(\theta ,\varphi ,t\right)-u_{L}{\left(\theta ,\varphi ,t\right)∥}_{L_{2}\left(\Omega \times S^{2}\right)}\leq C\exp\left(-\frac{c^{2}t}{2D}\right){\left(\sum_{l=L}^{\infty }\left(2l+1\right)C_{l}\right)}^{1/2}, \end{array}$$

see (36) in [8].

The following result shows that the considered model is more complex. In the general case of an arbitrary measure G(·) it is impossible to get a bound similar to (39) even for a sufficiently large *L*.

**Theorem** **10.** *For any fixed C>0 and L∈N there exist t>0 and an initial random condition $\eta \left(x\right),\;x\in R^{3}$, such that the norm of the approximation error ${\tilde{T}}_{H}\left(\theta ,\varphi ,t\right)-{\tilde{T}}_{H,L}\left(\theta ,\varphi ,t\right)$ does not satisfy* (39).

**Proof** **of** **Theorem** **10.** Indeed, let us consider some ε∈(0,1).

Then, $\sqrt{1-\frac{4D^{2}}{c^{2}}\mu^{2}}\geq 1-\varepsilon$ if $\mu \in I_{\varepsilon }:=\left[0,\sqrt{\frac{c^{2}}{4D^{2}}\left(1-{\left(1-\varepsilon \right)}^{2}\right)}\right]$.

Let the measure G(·) be concentrated on the interval $I_{\varepsilon }$. By (16), if $\mu \in I_{\varepsilon }$ then

$${\tilde{H}}_{1}\left(\mu ,t\right)\geq \exp\left(-\frac{c^{2}}{2D}t\left(1-\sqrt{1-\frac{4D^{2}}{c^{2}}\mu^{2}}\right)\right)\geq \exp\left(-\frac{c^{2}}{2D}t\varepsilon \right).$$

Hence, by (38) and Theorem 3 for any C,L>0, there exists t,ε>0, and the measure G(·) such that for the corresponding ${\tilde{T}}_{H}\left(\theta ,\varphi ,t\right)$ and ${\tilde{T}}_{H,L}\left(\theta ,\varphi ,t\right)$ it holds

$$\begin{array}{cc} ∥{\tilde{T}}_{H}\left(\theta ,\varphi ,t\right)-{\tilde{T}}_{H,L}{\left(\theta ,\varphi ,t\right)∥}_{L_{2}\left(\Omega \times S^{2}\right)} & \geq \frac{1}{2\sqrt{\pi }}\exp\left(-\frac{c^{2}}{2D}t\varepsilon \right){\left(\sum_{l=L}^{\infty }\left(2l+1\right)C_{l}\right)}^{1/2}\\ \; & \geq C\exp\left(-\frac{c^{2}}{2D}t\right){\left(\sum_{l=L}^{\infty }\left(2l+1\right)C_{l}\right)}^{1/2}. \end{array}$$

 □

However, it is possible to obtain a rate of convergence that is exponential in *t* if the measure G(·) has a bounded support.

**Theorem** **11.** *Let $\eta \left(x\right),\;x\in R^{3}$, have the measure G(·) such that G([0,δ])=0 for some $\delta \in (0,\frac{c}{2D})$. Then, for the solution ${\tilde{T}}_{H}\left(\theta ,\varphi ,t\right)$ of the initial value problem* (1) *and* (2) *and its approximation ${\tilde{T}}_{H,L}\left(\theta ,\varphi ,t\right)$ it holds that*

$$∥{\tilde{T}}_{H}\left(\theta ,\varphi ,t\right)-{\tilde{T}}_{H,L}{\left(\theta ,\varphi ,t\right)∥}_{L_{2}\left(\Omega \times S^{2}\right)}\leq C\exp\left(-D\delta^{2}t\right){\left(\sum_{l=L}^{\infty }\left(2l+1\right)C_{l}\right)}^{1/2}.$$

**Proof** **of** **Theorem** **11.** As $\frac{sinh(x)}{x}$ is an increasing function on (0, ∞) it follows from (16) that for μ≥δ

$${\tilde{H}}_{1}\left(\mu ,t\right)\leq \exp\left(-\frac{c^{2}}{2D}t\right)\left(\exp\left(\frac{c^{2}}{2D}t\sqrt{1-\frac{4D^{2}}{c^{2}}\delta^{2}}\right)+\exp\left(\frac{c^{2}}{2D}t\sqrt{1-\frac{4D^{2}}{c^{2}}\delta^{2}}\right)\frac{1}{\sqrt{1-\frac{4D^{2}}{c^{2}}\delta^{2}}}\right)$$

$$\begin{array}{cc} \; & \leq \exp\left(-\frac{c^{2}}{2D}t\left(1-\sqrt{1-\frac{4D^{2}}{c^{2}}\delta^{2}}\right)\right)\left(1+\frac{1}{\sqrt{1-\frac{4D^{2}}{c^{2}}\delta^{2}}}\right)\\ \; & =\left(1+\frac{1}{\sqrt{1-\frac{4D^{2}}{c^{2}}\delta^{2}}}\right)\exp\left(-\frac{c^{2}}{2D}t\times \frac{4D^{2}\delta^{2}}{c^{2}\left(1+\sqrt{1-\frac{4D^{2}}{c^{2}}\delta^{2}}\right)}\right)\\ \; & \leq \left(1+\frac{1}{\sqrt{1-\frac{4D^{2}}{c^{2}}\delta^{2}}}\right)\exp\left(-D\delta^{2}t\right). \end{array}$$

Notice that for x≥0 and a∈(0,1) it holds that $1+x\leq \frac{1}{a}\exp\left(xa\right)$.

Then, using the definition of ${\tilde{H}}_{2}\left(\mu ,t\right)$ in (17) we get for t≥0

$$\begin{array}{cc} {\tilde{H}}_{2}\left(\mu ,t\right) & \leq \exp\left(-\frac{c^{2}}{2D}t\right)\left(1+\frac{c^{2}}{2D}t\right)\leq \exp\left(-\frac{c^{2}}{2D}t\right)\frac{1}{\sqrt{1-\frac{4D^{2}}{c^{2}}\delta^{2}}}\exp\left(\frac{c^{2}}{2D}t\sqrt{1-\frac{4D^{2}}{c^{2}}\delta^{2}}\right)\\ \; & \leq \frac{1}{\sqrt{1-\frac{4D^{2}}{c^{2}}\delta^{2}}}\exp\left(-D\delta^{2}t\right). \end{array}$$

Hence, if G([0,δ])=0 it follows from Theorem 3 that

$$C_{l}\left(t,t\right)\leq {\left(1+\frac{1}{\sqrt{1-\frac{4D^{2}}{c^{2}}\delta^{2}}}\right)}^{2}\exp\left(-2D\delta^{2}t\right)C_{l}.$$

Applying this bound to (38) we obtain the statement of the theorem. □

The next result follows from (38) and the upper bound (19) for ${\tilde{H}}_{2}\left(\mu ,t\right)$.

**Corollary** **1.** *If $G\left([0,\frac{c}{2D}]\right)=0$, then*

$$∥{\tilde{T}}_{H}\left(\theta ,\varphi ,t\right)-{\tilde{T}}_{H,L}{\left(\theta ,\varphi ,t\right)∥}_{L_{2}\left(\Omega \times S^{2}\right)}\leq \frac{1}{2\sqrt{\pi }}\left(1+\frac{c^{2}}{2D}t\right)\exp\left(-\frac{c^{2}}{2D}t\right){\left(\sum_{l=L}^{\infty }\left(2l+1\right)C_{l}\right)}^{1/2}.$$

**Remark** **4.** *The rates of convergence in Theorems* 9, 11 *and Corollary* 1 *are sharp. Indeed, for t=0 one obtains*

$$\begin{array}{cc} ∥{\tilde{T}}_{H}\left(\theta ,\varphi ,0\right)-{\tilde{T}}_{H,L}{\left(\theta ,\varphi ,0\right)∥}_{L_{2}\left(\Omega \times S^{2}\right)} & ={\left(\sum_{l=L}^{\infty }\sum_{m=-l}^{l}Y_{lm}\left(\theta ,\varphi \right)Y_{lm}^{*}\left(\theta ,\varphi \right)C_{l}\left(0,0\right)\right)}^{1/2}\\ \; & =\frac{1}{2\sqrt{\pi }}{\left(\sum_{l=L}^{\infty }\left(2l+1\right)C_{l}\right)}^{1/2}. \end{array}$$

The angular power spectrum $\{C_{l}$, l=0,1,⋯} of the initial random field η(x) is determined by the measure G(·). The following results provide some insight into the behaviour of $\sum_{l=L}^{\infty }\left(2l+1\right)C_{l}$ in terms of the spectral measure G(·).

**Theorem** **12.** *Let the angular power spectrum of η(x) be $\{C_{l}$, l=0,1,⋯}.*

(a) *Then, it holds that*
  (40) $$\begin{array}{c} \sum_{l=L}^{\infty }\left(2l+1\right)C_{l}=2\pi^{2}\int_{0}^{\infty }\mu \left(J_{L-\frac{1}{2}}\left(\mu \right)J_{L+\frac{1}{2}}^{{}^{\prime }}\left(\mu \right)-J_{L+\frac{1}{2}}\left(\mu \right)J_{L-\frac{1}{2}}^{{}^{\prime }}\left(\mu \right)\right)G\left(d\mu \right). \end{array}$$
(b) *If $\int_{0}^{\infty }\mu^{1/3}G\left(d\mu \right)<\infty$, then*
  (41) $$\begin{array}{c} \sum_{l=L}^{\infty }\left(2l+1\right)C_{l}\leq C\int_{0}^{\infty }\frac{\mu G(d\mu )}{{\left(1+{\left(L-\frac{3}{2}\right)}^{2}+\mu^{2}\right)}^{1/3}},\;L\geq 2. \end{array}$$
(c) *If the measure G(·) has a bounded support [0,δ], δ>0, then*
  (42) $$\begin{array}{c} \sum_{l=L}^{\infty }\left(2l+1\right)C_{l}\leq \frac{C}{\Gamma^{2}\left(L-\frac{1}{2}\right)}{\left(\frac{\delta }{2}\right)}^{2L},\;L\geq 2. \end{array}$$

**Proof** **of** **Theorem** **12.** (a) It follows from the representation

$$C_{l}=2\pi^{2}\int_{0}^{\infty }\frac{J_{l+\frac{1}{2}}^{2}\left(\mu \right)}{\mu }G\left(d\mu \right)$$

that

(43) $$\begin{array}{c} \sum_{l=L}^{\infty }\left(2l+1\right)C_{l}=2\pi^{2}\int_{0}^{\infty }\sum_{l=L}^{\infty }\left(2l+1\right)J_{l+\frac{1}{2}}^{2}\left(\mu \right)\frac{G(d\mu )}{\mu }. \end{array}$$

By von Lommel’s formula (see (2.60) in [29]),

$$\sum_{n=0}^{\infty }\left(\nu +1+2n\right)J_{\nu +1+2n}^{2}\left(\mu \right)=\frac{\mu^{2}}{4}\left(J_{\nu }^{2}\left(\mu \right)-J_{\nu -1}\left(\mu \right)J_{\nu +1}\left(\mu \right)\right),$$

where μ∈R and ν>−1, we obtain

(44) $$\begin{array}{cc} \sum_{l=L}^{\infty }\left(2l+1\right)J_{l+\frac{1}{2}}^{2}\left(\mu \right) & =2\sum_{n=0}^{\infty }\left(L+\frac{1}{2}+2n\right)J_{L+\frac{1}{2}+2n}^{2}\left(\mu \right)+2\sum_{l=L}^{\infty }\left(L+1+\frac{1}{2}+2n\right)J_{L+1+\frac{1}{2}+2n}^{2}\left(\mu \right)\\ \; & =\frac{1}{2}\mu^{2}\left(J_{L-\frac{1}{2}}^{2}\left(\mu \right)-J_{L-\frac{3}{2}}\left(\mu \right)J_{L+\frac{1}{2}}\left(\mu \right)+J_{L+\frac{1}{2}}^{2}\left(\mu \right)-J_{L-\frac{1}{2}}\left(\mu \right)J_{L+\frac{3}{2}}\left(\mu \right)\right)\\ \; & =\frac{1}{2}\mu^{2}\left(J_{L-\frac{1}{2}}\left(\mu \right)\left(J_{L-\frac{1}{2}}\left(\mu \right)-J_{L+\frac{3}{2}}\left(\mu \right)\right)+J_{L+\frac{1}{2}}\left(\mu \right)\left(J_{L+\frac{1}{2}}\left(\mu \right)-J_{L-\frac{3}{2}}\left(\mu \right)\right)\right)\\ \; & =\mu^{2}\left(J_{L-\frac{1}{2}}\left(\mu \right)J_{L+\frac{1}{2}}^{{}^{\prime }}\left(\mu \right)-J_{L+\frac{1}{2}}\left(\mu \right)J_{L-\frac{1}{2}}^{{}^{\prime }}\left(\mu \right)\right). \end{array}$$

Now, (40) follows by substituting the last expression in (43).

(b) Using the inequality from [30]

$$|J_{\nu }\left(\mu \right)|\leq \frac{C}{{\left(1+\nu^{2}+\mu^{2}\right)}^{1/6}}$$

we obtain that for L≥2

$$|J_{L-\frac{1}{2}}\left(\mu \right)\left(J_{L-\frac{1}{2}}\left(\mu \right)-J_{L+\frac{3}{2}}\left(\mu \right)\right)+J_{L+\frac{1}{2}}\left(\mu \right)\left(J_{L+\frac{1}{2}}\left(\mu \right)-J_{L-\frac{3}{2}}\left(\mu \right)\right)|\leq 4\frac{C}{{\left(1+{\left(L-\frac{3}{2}\right)}^{2}+\mu^{2}\right)}^{1/6}},$$

which after the substitution in (43) gives (41).

(c) By the Poisson integral formula and the identity $\int_{0}^{1}{\left(1-t^{2}\right)}^{n}dt=\frac{\sqrt{\pi }\Gamma \left(n+1\right)}{2\Gamma (n+\frac{3}{2})}$ one obtains

(45) $$\begin{array}{c} |J_{L-\frac{3}{2}}\left(\mu \right)|\leq \frac{2{\left(\mu /2\right)}^{L-\frac{3}{2}}}{\sqrt{\pi }\Gamma \left(L-1\right)}\int_{0}^{1}{\left(1-t^{2}\right)}^{L-2}dt=\frac{{\left(\mu /2\right)}^{L-\frac{3}{2}}}{\Gamma (L-\frac{1}{2})}. \end{array}$$

If [0,δ],δ>0, is the support of the measure G(·), then it follows from (43)–(45) that

$$\sum_{l=L}^{\infty }\left(2l+1\right)C_{l}\leq \frac{C}{2^{2L-3}\Gamma^{2}\left(L-\frac{1}{2}\right)}\int_{0}^{\delta }\max\left(\mu^{2(L-\frac{3}{2})+1},\mu^{2(L+\frac{3}{2})+1}\right)G\left(d\mu \right),$$

which completes the proof. □

## 7. Numerical Studies

This section presents numerical studies of the solution $T_{H}\left(x,t\right)$, its angular spectrum, and covariance functions over time. We also provide some numerical analysis of approximation errors.

All numerical computations and simulations in this paper were performed using the software R version 3.6.1 and Python version 3.7.5. The results were derived using the HEALPix representation of spherical data (see [31] and http://healpix.sourceforge.net). In particular, the R package rcosmo [32,33] was used for computations and visualisations of the obtained results. The Python package healpy was used for fast spherical harmonics generation of spherical maps from Laplace series coefficients. The R and Python code used for numerical examples in Section 7 are freely available in the folder “Research materials” from the website https://sites.google.com/site/olenkoandriy/.

It is important to clarify that the numerical analysis in this paper is rather different from the one in [8] and requires more advanced approximation approaches. Namely, the stochastic model in [8] yielded the representation of the Laplace series coefficients $a_{lm}\left(t\right)=C\left[A_{l}\left(t\right)+B_{l}\left(t\right)\right]a_{lm}\left(0\right)$ for some functions $A_{l}\left(t\right)$ and $B_{l}\left(t\right)$ which can be explicitly computed in terms of elementary functions. However, for the model (1) and (2) there is no such simple functional relation that links $a_{lm}\left(t\right)$ and $a_{lm}\left(0\right)$. As a result, there are no explicit elementary functional relations between $C_{l}\left(t,t^{\prime }\right)$, $R(\cos\gamma ,t,t^{\prime })$ and $C_{l}\left(0,0\right)$, R(cosγ,0,0) respectively. To compute spectral and covariance functions of $T_{H}\left(x,t\right)$ at time t>0 one has to use formulae (26), (28) and (29). These integral representations are given in terms of the spectral measure G(·) and stochastic measures $Z_{lm}\left(\cdot \right)$ of the initial random condition field η(x).

By (1.2.5) in [26], it follows from

$$R\left(r\right)=\int_{0}^{\infty }\frac{\sin(\mu r)}{\mu r}G\left(d\mu \right)$$

that

(46) $$\begin{array}{c} G\left(\mu \right)=\sqrt{\frac{2}{\pi }}\int_{0}^{\infty }J_{3/2}\left(ur\right){\left(ur\right)}^{3/2}\frac{R(r)}{r}dr, \end{array}$$

which can be used to compute (26), (29) and simulate $Z_{lm}\left(\cdot \right)$ for computations in (28). However, obtaining a reliable approximation of the integral in (46) and stochastic measures $Z_{lm}\left(\cdot \right)$ requires the estimation of the empirical covariance function $\hat{R}\left(r\right)$ on a dense grid. Moreover, for data observed on bounded subsets of $R^{3},$ covariance functions can be estimated only for distances that do not exceed their diameters. Thus, it is important to verify that empirical covariance functions are sufficiently quickly decaying to be assumed negligible for distances greater than these diameters. We postpone the solution of these technical problems and analysis of real data to future publications.

**Figure 1:**
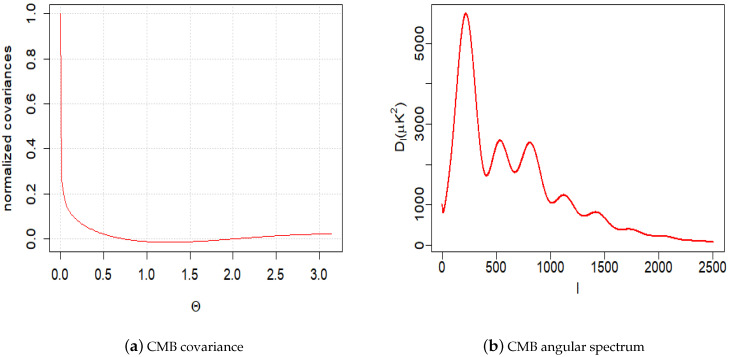
CMB: Cosmic Microwave Background radiation.

In the following examples, we study the properties of solutions and their approximations using simulated data. The examples were constructed to demonstrate that the model is sufficiently powerful to imitate behaviours of the empirical CMB covariance function and oscillating angular spectrum (see [8,34]). The actual CMB covariance function and angular spectrum are shown in Figure 1a,b. Note that the estimated angular CMB spectrum shown in Figure 1b was obtained by a piecewise fitting of several physical models and interpolation techniques for different intervals of the spectrum [6,13]. Some actual spectrum estimates deviate substantially from the fitted curve in Figure 1b [34]. Therefore, in predicting CMB and spectrum changes over time, small details can be ignored and one needs to focus on a general pattern. Thus, the following examples with the analysis of simulated data which spectrum is analogous to the real one can offer important insights on the future evolution of CMB and its spectral properties.

In the examples, the case of a discrete measure G(·) is considered (i.e., the support of G(·) is a finite set $\left\{\mu_{i},\;i=1,\cdots ,I\right\}$). We employ real-valued stochastic measures $Z_{lm}\left(\cdot \right)$ that are concentrated on this set and satisfy the condition

$$G\left(\mu_{i}\right)=E\;Z_{lm}^{2}\left(\mu_{i}\right)=\sigma_{i}^{2},\;i=1,\cdots ,I.$$

We assume that the random field η(x) is centred Gaussian. Hence, we can choose $Z_{lm}\left(\mu_{i}\right)∼N\left(0,\sigma_{i}^{2}\right)$ that are independent for different *l*, *m* and *i*.

In these settings, formulae (26), (28) and (29) take the following discrete forms:

(47) $$R\left(\cos\gamma ,t,t^{\prime }\right)=\sum_{i=1}^{I}\frac{\sin(2\mu_{i}\sin\left(\frac{\gamma }{2}\right))}{2\mu_{i}\sin\left(\frac{\gamma }{2}\right)}\tilde{H}\left(\mu_{i},t\right)\tilde{H}\left(\mu_{i},t^{\prime }\right)\sigma_{i}^{2},$$

$$a_{lm}\left(t\right)=\pi \sqrt{2}\sum_{i=1}^{I}\frac{J_{l+\frac{1}{2}}\left(\mu_{i}\right)}{\sqrt{\mu_{i}}}\tilde{H}\left(\mu_{i},t\right)Z_{lm}\left(\mu_{i}\right),$$

(48) $$\begin{array}{c} C_{l}\left(t,t^{\prime }\right)=2\pi^{2}\sum_{i=1}^{I}\frac{J_{l+\frac{1}{2}}^{2}\left(\mu_{i}\right)}{\mu_{i}}\tilde{H}\left(\mu_{i},t\right)\tilde{H}\left(\mu_{i},t^{\prime }\right)\sigma_{i}^{2}, \end{array}$$

which are convenient for simulations.

This approach can also be used to approximate absolutely continuous spectral measures G(·) by considering a sufficiently large *I*, small $|\mu_{i}-\mu_{i+1}|$ and $\sigma_{i}^{2}=G\left([\mu_{i},\mu_{i+1}]\right)$, i=1,...,I.

**Example** **1.** *This example illustrates changes over time of the covariance function R(cosγ,0,t) defined by (26) and the power spectrum $C_{l}\left(t,t\right)$ defined by (29). To produce plots and computations we used the corresponding discrete Equations (47) and (48) with values $\sigma_{i}=\frac{100}{i}$ by i∈{1,2,⋯,10} and a discrete spectrum concentrated on the interval [1,40]. All computations and plots in this example are presented for the values c=1 and D=1 of the parameters in Equation (1).*

**Figure 2:**
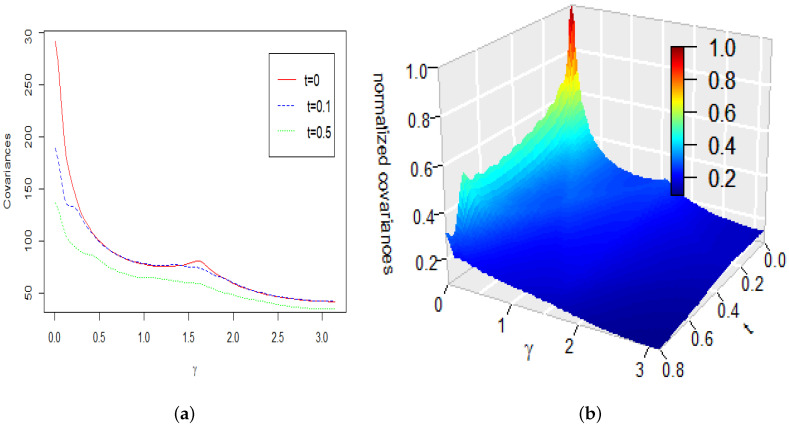
(**a**) R(cosγ,t,t) at the time lags t=0,0.1 and 0.5 and angular distances γ for c=D= 1. (**b**) R(cosγ,t,t) for c=D=1 at time lag t and angular distance γ.

*Figure 2a shows the covariance R(cosγ,t,t) at the time lags t=0,t=0.1 and t=0.5 as functions of the angular distance γ. To understand the effect of time and the angular distance γ on the covariance function we provided 3D-plots (see Figure 2b) showing the covariance as a function of the time lag t. The plot in Figure 2b is normalized by dividing each value by $\max_{\gamma \in [0,\pi ]}R\left(\cos\gamma ,0,0\right)$. It is obvious that the covariance decays through time and changes very little except for values of γ, which are close to 0.*

**Figure 3:**
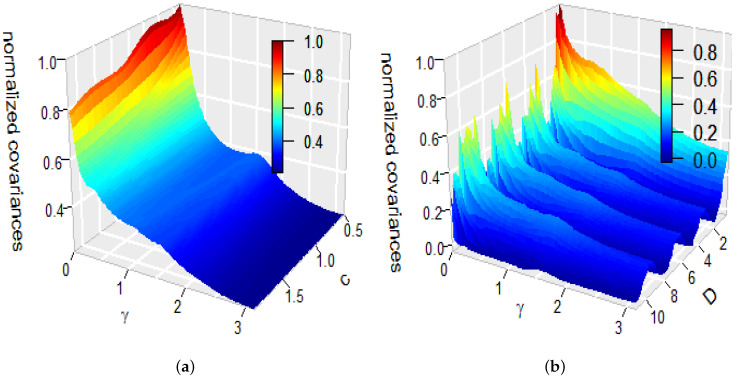
(**a**) R(cosγ,0.1,0.1) as a function of γ and c for D=1. (**b**) R(cosγ,0.1,0.1) as a function of γ and D for c=1.

*To understand the effect of the parameters c and D on the covariance function, we also produced Figure 3. It illustrates changes of the covariance function R(cosγ,t,t) at a specific time t as functions of the angular distances γ and the parameters c or D. To produce this figure we used t=0.1. Figure 3a displays R(cosγ,0.1,0.1) for D=1 as a function of c and the angular distances γ. Figure 3b displays R(cosγ,0.1,0.1) for c=1 as a function of D and the angular distances γ. The plots in Figure 3 are normalised by dividing each value by $\max_{\gamma \in [0,\pi ]}R\left(\cos\gamma ,0.1,0.1\right)$. It is clear form Figure 3a that the covariance decays through c (also through D; see Figure 3b) and changes very little except values of γ which are close to 0. Figure 3b demonstrates that the normalised covariance function exhibits decaying periodic behaviour when D increases.*

**Figure 4:**
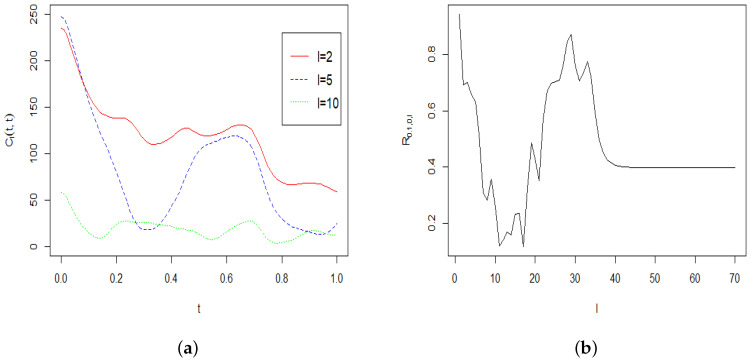
(**a**) The power spectrum $C_{l}\left(t,t\right)$ for c=D=1 and values l=2,5 and 10. (**b**) The ratio $R_{0.1,0,l}$ of the first 70 coefficients for c=D= 1.

*Figure 4a displays the power spectrum $C_{l}\left(t,t\right)$ as a function of t≥0. To produce this figure we used t∈[0,1] and l=2,5 and 10. The first 70 coefficients $C_{l}$ were computed by applying the Equation (48) with the above values of $\sigma_{i},\;i=1,\cdots ,10$. From this figure it is clear that the power spectrum $C_{l}\left(t,t\right)$ decays very quickly to 0 when l increases. To investigate the effect of the parameter l we provide a plot of the ratio $R_{0.1,0,l}=C_{l}\left(0.1,0.1\right)/C_{l}\left(0,0\right)$ for the first 70 coefficients $C_{l}$ (see Figure 4b). This figure confirms that the ratio $R_{0.1,0,l}$ is bounded by 1 and changes very little when l increases.*

**Figure 5:**
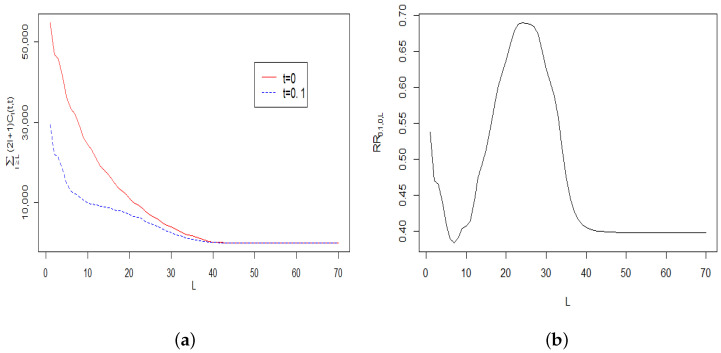
(**a**) Plots of $\sum_{l\geq L}\left(2l+1\right)C_{l}\left(t,t\right)$ at t=0 and t=0.1. (**b**) The ratio $RR_{0.1,0,L}$.

*Figure 5a plots the tail sums $\sum_{l\geq L}\left(2l+1\right)C_{l}\left(0,0\right)$ and $\sum_{l\geq L}\left(2l+1\right)C_{l}\left(0.1,0.1\right)$ as functions of L, while Figure 5b displays the corresponding ratio $RR_{0.1,0,L}=\frac{\sum_{l\geq L}\left(2l+1\right)C_{l}\left(0.1,0.1\right)}{\sum_{l\geq L}\left(2l+1\right)C_{l}\left(0,0\right)}$. From Figure 5a it is clear that when L increases, both the terms $\sum_{l\geq L}\left(2l+1\right)C_{l}\left(0,0\right)$ and $\sum_{l\geq L}\left(2l+1\right)C_{l}\left(0.1,0.1\right)$ have the same asymptotic behaviour up to a constant multiplier, which is also further confirmed in Figure 5b.*

**Example** **2.** *In this example we use a discrete spectrum concentrated on the two intervals [0,20] and [80,90]. Thus, the initial condition random field η(x) has low- and high-frequency components. To produce realisations of η(x) and $T_{H}\left(x,t\right),\;x\in S^{2},$ which are similar to small real CMB values, we used $\sigma_{i}^{2}=0.00003$ and 0.0001 for low- and high-frequency components, respectively. These small values allow us to employ the visualisation tools and colour palettes used for CMB plotting in the R package rcosmo [33] and the Python package healpy.*

**Figure 6:**
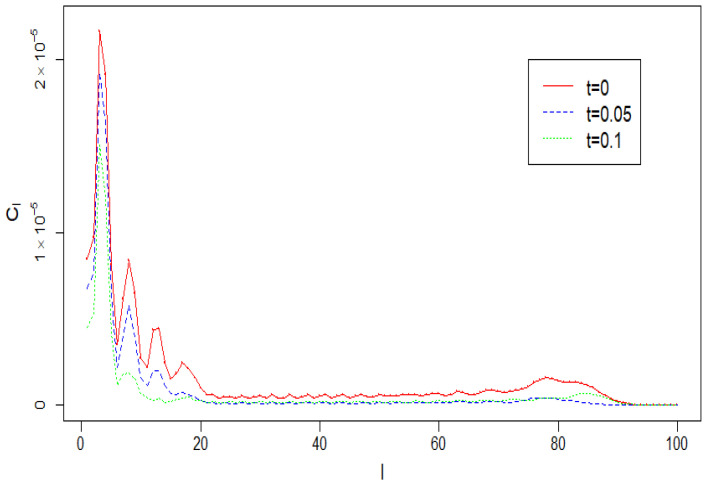
Angular power spectra $C_{l}\left(t,t\right)$ for c=1 and D=2 at time t=0,0.05 and 0.1.

**Figure 7:**
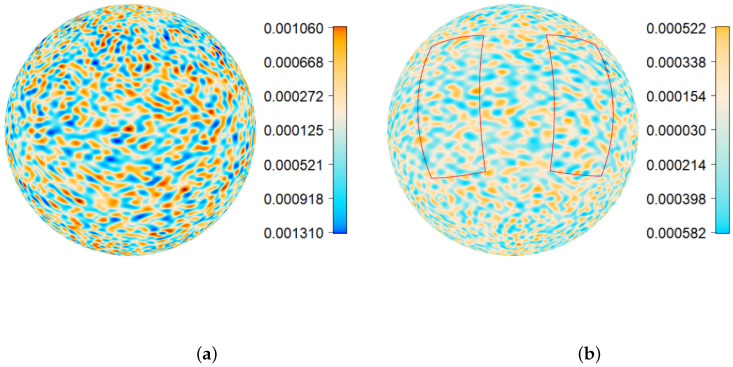
(**a**) Realisation of $T_{H}\left(\theta ,\varphi ,0\right)$ for c=1 and D=2. (**b**) Realisation of $T_{H}\left(\theta ,\varphi ,0.05\right)$ for c=1 and D=2 with two observation windows.

*To produce the plots and computations in this paper we used the first 100 coefficients $C_{l}$ obtained by applying (48) to the above discrete spectrum. They are shown in Figure 6 in red. In this example we use the values c=1 and D=2 of the parameters in Equation (1). The coefficients $C_{l}\left(t,t\right)$ for t=0.05 and 0.1 are plotted in blue and green respectively. The graph indicates two regions with relatively large values of $C_{l}$ that correspond to the spectral measure G(·) used for these computations. It can be seen that values $C_{l}\left(t,t\right)$ decrease over time. However, the corresponding spherical maps change rather slowly. Therefore, only two maps, for t=0 and 0.05, are plotted in Figure 7.*

**Figure 8:**
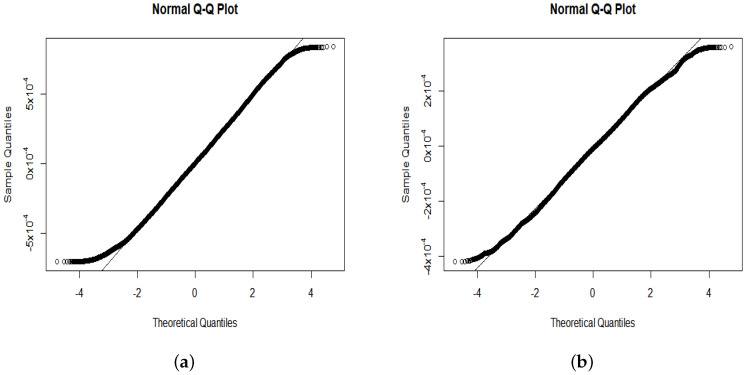
(**a**) Normal Q–Q plot of all $T_{H}\left(\theta ,\varphi ,0\right)$ values from window 2 in Figure 7b. (**b**) Normal Q–Q plot of all $T_{H}\left(\theta ,\varphi ,0.05\right)$ values from window 2 in Figure 7b.

**Figure 9:**
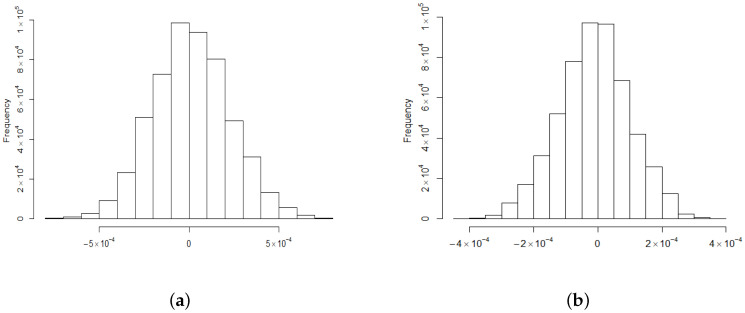
(**a**) Histogram of all $T_{H}\left(\theta ,\varphi ,0\right)$ values from window 2 in Figure 7b. (**b**) Histogram of all $T_{H}\left(\theta ,\varphi ,0.05\right)$ values from window 2 in Figure 7b.

*For the following numerical studies we used simulated data from two windows shown in Figure 7b. The estimated means in Table 1 confirm that $T_{H}\left(\theta ,x,t\right)$ has a zero mean. It can be observed from Figure 7 and the estimated interquartile ranges (IQRs) in Table 1 that the magnitude of $T_{H}\left(x,t\right)$ values decrease with time. However, the distribution type of the combined values does not change substantially. Namely, the combined values of $T_{H}\left(x,t\right)$ exhibit an approximately bell-shaped behaviour with tails that are heavier than in the Gaussian case (see Figure 8 and Figure 9). Similar results were obtained for various observation windows of $S^{2}$. For example, for the second rectangular window shown in Figure 7b Q–Q plots and histograms of observations in this window are given in Figure 8 and Figure 9, respectively. These results about distributions of combined values were also confirmed by computing the Shannon entropy*

$$\hat{H}=-\sum_{i=1}{\hat{p}}_{i}\log\left({\hat{p}}_{i}\right)$$

*for the empirical distributions $\left\{\hat{p_{i}}\right\}$ given by the histograms in Figure 9. Values of $\hat{H}$ do not change much over time t (Table 1). They are not substantially different from the entropy upper bound log(16)≈2.77.*

*The q-statistic, see [35], was used to investigate heterogeneity between values of $T_{H}\left(\theta ,\varphi ,t\right)$ in windows 1 and 2 from Figure 7b. Table 1 indicates that heterogeneity is absent at time 0 and the evolution due to the model (1) does not introduce heterogeneity, at least for short time periods.*

## 8. Entropy and Hyperbolic Diffusion

This section discusses the evolution of Shannon entropy for hyperbolic diffusion. Theoretical analysis and several numerical examples are presented. To simplify the exposition and plots, only the case of x∈R and various non-random initial conditions are studied.

For diffusive transport that arises from the random motion of particles, the mass distribution may indeed be regarded as a probability distribution, after which the Shannon entropy may be calculated. For a simple thermodynamic system governed purely by linear or nonlinear heat conduction, there is a close analogy between thermodynamic entropy and Shannon entropy (e.g., [24,36]). When the transport mechanism is modified to hyperbolic diffusion, the behaviour of entropy requires more scrutiny. In order to illustrate this, consider one-dimensional solutions q(x,t) on $\left[-\ell ,\ell \right]\times R^{+}$, subject to Neumann boundary conditions

$$q_{x}\left(x,t\right)=0,\;\;\;x=\pm L.$$

This may represent transport in the *x*-direction through a linear conduit of cross-sectional area *A*, with the variation of density in each cross section being effectively zero. It will be seen that the total mass *M* is constant. Therefore, the scaled density $q^{*}=qA/M$ has constant unit integral on [−L,L], from which physically relevant non-negative solutions $q^{*}\left(x,t\right)$ may be regarded as distributions. By choosing length scale D/c and time scale $D/c^{2}$, it may be assumed that the coefficients in the hyperbolic diffusion equation are normalised to ±1.

Let $t*=tc^{2}/D$, $x^{*}=xc/D$ and $L^{*}=Lc/D$. Then,

$$q_{t^{*}}^{*}+q_{t^{*}t^{*}}^{*}=q_{x^{*}x^{*}}^{*},$$

subject to boundary conditions

$$q_{x^{*}}^{*}=0,\;\;x^{*}=\pm L^{*}$$

and initial conditions

$$q^{*}\left(x^{*},0\right)=u_{0}\left(x^{*}\right),\;\;q_{t^{*}}^{*}\left(x^{*},0\right)=v_{0}\left(x^{*}\right).$$

Defining Shannon entropy density to be $s=-q^{*}\log q^{*}$, the hyperbolic diffusion equation for $q^{*}\left(x,t\right)$ implies

(49) $$s_{t}+\frac{D}{c^{2}}s_{tt}=D\frac{q_{x}^{*2}-\frac{1}{c^{2}}q_{t}^{*2}}{q^{*}}.$$

The case of unbounded speed of propagation is obtained by taking the limit c→∞, which results in a positive entropy production rate $Dq_{x}^{*2}/q^{*}$. This is familiar from the theory of heat conduction, for which the entropy production rate is $L_{e}DT_{x}^{2}/T$, where *T* is absolute temperature and $L_{e}$ is the Lewis number, which is the order-1 ratio of thermal diffusivity to mass diffusivity.

For uni-directional waves of velocity ±c, the entropy production rate is zero. For bi-directional waves, the total Shannon entropy is constant when opposite-travelling waves are not superposing, increasing when opposite-travelling superposing waves are separating, and decreasing when they are superposing and approaching. However, non-constant travelling wave solutions of the hyperbolic diffusion equation must have speed less than *c* and they must have an amplitude that decreases with time. For the remainder of this section, the asterisk superscripts will be conveniently omitted.

Some solutions of the hyperbolic diffusion equation may be of dissipative diffusive type, while others may be dissipative bi-directional waves. In order to illustrate this, by the completeness of the Fourier transform, the general even solution by the separation of variables is

(50) $$\begin{array}{c} q=a_{0}+\sum_{n=1}^{n_{c}}\left[a_{n}e^{-\alpha_{n}^{+}t}+b_{n}e^{\alpha_{n}^{-}t}\right]\cos\left(k_{n}x\right) \end{array}$$

(51) $$\begin{array}{c} +\sum_{n=n_{c}+1}^{\infty }a_{n}e^{-0.5t}\cos\left(\omega_{n}t\right)\cos\left(k_{n}x\right), \end{array}$$

where $n_{c}={\left[L/2\pi \right]}_{-}$, $k_{n}=n\pi /L$, $\omega_{n}=k_{n}\sqrt{1-1/{\left(2k_{n}\right)}^{2}}$ and $\alpha_{n}^{\pm }=\frac{1}{2}\left(1\pm \sqrt{1-4k_{n}^{2}}\right)$.

The first summation covers modes that are purely dissipative in character, just as for the linear heat diffusion equation. However, in this case, the dissipative modes exist only when L≥2π. The second summation covers standing wave modes with decaying amplitude. These may be regarded as a superposition of a decaying left-travelling wave and a decaying right-travelling wave. Note that the dissipative mode with logarithmic decay rate $\alpha_{1}^{-}$ decays more slowly than all other modes.

The above solution is mass-conserving with mean value $a_{0}$ and constant mass integral $2La_{0}=1$ by normalisation. For a single decaying standing wave mode of a hyperbolic diffusion equation distribution, for some value of *t*,

$$q=\frac{1}{2L}\left[1+e^{-0.5t}\cos\left(\omega_{n}t\right)\cos\left(k_{n}x\right)\right].$$

Then, the total Shannon entropy is

$$S=\int_{-L}^{L}q\log\left(1/q\right)dx.$$

At times $t=\left(2m+1\right)\pi /2\omega_{n},\;\;m\in Z$, the distribution is uniform, which is the state of maximum entropy S=log(2L). Overall, the total entropy oscillates as it approaches the limiting equilibrium state. However the negative excursions of entropy may be quite small since the amplitude of oscillation decreases exponentially.

**Figure 10:**
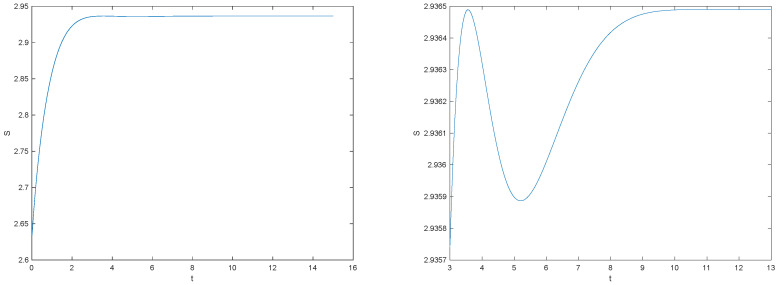
Total entropy for standing wave with single harmonic. L=3π, wave number $k_{2}=2\pi /L.$

Figure 10 plots the total entropy for a wave with single harmonic, calculated by trapezoidal integration with 400 intervals, versus time.

**Figure 11:**
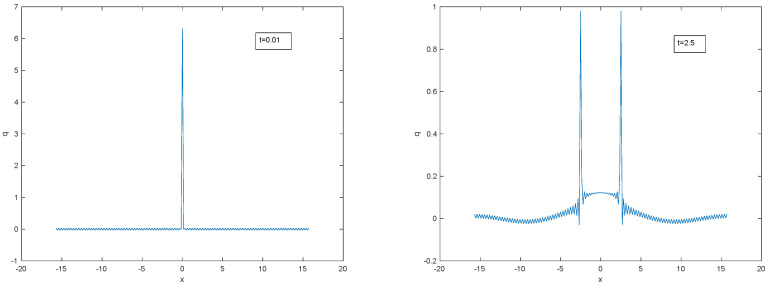
Evolving spike solution for L=3π.

It would be helpful to have a point-source solution for the hyperbolic diffusion equation. As far as we are aware, there is no known simple expression for the point source evolution but it has the standard uniform Fourier spectrum that evolves according to (50). It is plotted in Figure 11 after truncating the Fourier series at 100 terms. As in the d’Alembert wave equation, two separating travelling delta waves emerge but now the amplitudes of the truncated spikes are decreasing and there is an additional central symmetric hump due to the purely diffusive terms. The leading edges of the spikes are travelling at maximum speed *c*. In two and three dimensions there would be similar solutions with a single travelling cylindrical or spherical shock wave surrounding a central hump.

**Figure 12:**
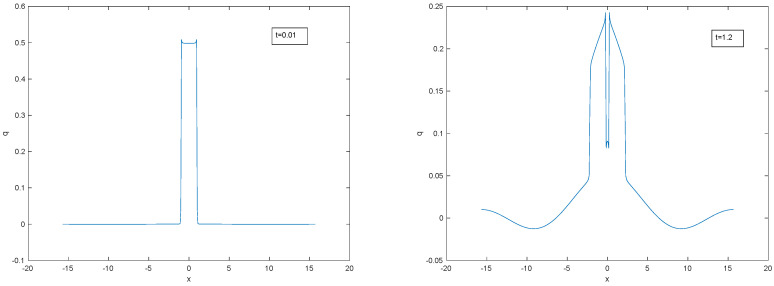
Evolving symmetric rectangle: emergent bidirectional wave.

**Figure 13:**
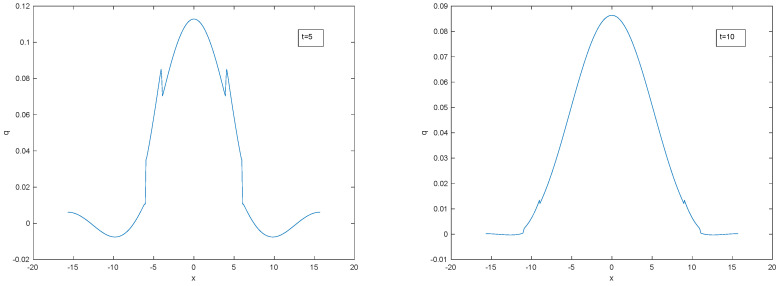
Evolving rectangle: dominant diffusive hump at large *t*, with leading edge of remnant rectangle demarcating the extent of the disturbance.

It is instructive also to view the motion of an initial rectangular disturbance of finite amplitude. This is approximated in Figure 12 and Figure 13 by a Fourier series of 200 terms. The truncated Fourier series is an exact solution, but due to the truncation and the boundary conditions, the solution is negative at some values of the domain, so that Shannon entropy cannot be calculated. However, the solution is indicative of the behaviour of a non-negative solution with initial rectangle. As in the bidirectional wave equation, the symmetric solution consists of two superposed rectangles that increase entropy as they begin to separate by travelling in opposite directions. After they have separated, their amplitude decreases, which leads to further entropy increase. The height of the leading edge decreases more rapidly than the trailing edge, so each rectangle evolves to a trapezoid. The leading edge—which is the boundary of the disturbance—continues to move at maximum speed *c*. Between the trapezoids, there is a central hump that eventually dominates, and resembles a diffusive Gaussian, increasing entropy further. With this kind of peaked initial condition, there is no indication of any significant period of entropy decrease.

## 9. Future Research Problems

This paper investigated evolutions of random fields determined by hyperbolic diffusion equations with random initial conditions. Spherical random fields were modelled as restrictions of 3D solution fields to the sphere. Compared to the previous publications, it resulted in a more realistic physical model. However, the solution field for the new model cannot be represented by using Laplace series coefficients $a_{lm}\left(0\right)$ of the initial condition directly. The more complicated representation involves spectral measures of initial random conditions.

Detailed studies of the solutions and their approximations were presented. In particular, regularity properties and temporal dependencies of solutions were investigated. Approximations to the SPDE solutions were proposed, and the upper bound analysis of approximation errors was provided. It was demonstrated that the magnitude of approximation errors is determined by the angular power spectrum $C_{l}$ and decreases at the rate of the cumulative tail sums ${\left(\sum_{l=L}^{\infty }\left(2l+1\right)C_{l}\right)}^{1/2}.$

The numerical studies investigated the dependence of solution fields on parameters of the SPDE model and provided some insight into the evolution of Shannon entropy for hyperbolic diffusion.

Some important problems and extensions for future research are:

- Investigating the sharpness of the obtained upper bounds on approximation errors (see [8]);
- Developing statistical estimators of the equation parameters and studying their asymptotic properties;
- Extending the methodology to tangent spherical vector fields (see [37]);
- Developing numerical methods for the obtained representations to deal with spectra of initial conditions;
- Extending the analysis and numerical studies in Section 8 to other scenarios;
- In line with the theme of this Special Issue, in future we intend to study the effect of nonlinear diffusivity in the equation
  $$q_{t}+\frac{1}{c^{2}}q_{tt}=\nabla \cdot \left[D\left(q\right)\nabla q\right].$$
  For example, if *q* is the electron density in a plasma, D(q) is typically decreasing [38].

## Figures and Tables

**Table 1:** Statistics for windows 1 and 2.

Time	0	0.05	10
Mean for window 1	$1.353\times 10^{-5}$	$-5.62\times 10^{-6}$	$3.501\times 10^{-7}$
Mean for window 2	$7.083\times 10^{-6}$	$-1.132\times 10^{-5}$	$-5.166\times 10^{-8}$
IQR for window 1	$2.877\times 10^{-4}$	$1.307\times 10^{-4}$	$6.78\times 10^{-6}$
IQR for window 2	$3.252\times 10^{-4}$	$1.452\times 10^{-4}$	$7.11\times 10^{-6}$
Entropy for window 1	2.193	2.116	2.369
Entropy for window 2	2.302	2.221	2.387
*q*-statistics	$1.986\times 10^{-4}$	$7.272\times 10^{-4}$	$1.5\times 10^{-3}$
